# *Bacillus* VOCs in the Context of Biological Control

**DOI:** 10.3390/antibiotics12030581

**Published:** 2023-03-15

**Authors:** Jovana Grahovac, Ivana Pajčin, Vanja Vlajkov

**Affiliations:** Faculty of Technology Novi Sad, University of Novi Sad, Bulevar cara Lazara 1, 21000 Novi Sad, Serbia

**Keywords:** antibacterial, antifungal, biocontrol agent, mechanism of action, microbial biopesticides, nematicidal, plant disease, plant pathogen, sustainable agriculture

## Abstract

A contemporary agricultural production system relying on heavy usage of agrochemicals represents a questionable outlook for sustainable food supply in the future. The visible negative environmental impacts and unforeseen consequences to human and animal health have been requiring a shift towards the novel eco-friendly alternatives for chemical pesticides for a while now. Microbial-based biocontrol agents have shown a promising potential for plant disease management. The bacteria of the genus *Bacillus* have been among the most exploited microbial active components due to several highly efficient mechanisms of action against plant pathogens, as well as a palette of additional plant-beneficial mechanisms, together with their suitable properties for microbial biopesticide formulations. Among other bioactive metabolites, volatile organic compounds (VOCs) have been investigated for their biocontrol applications, exhibiting the main advantage of long-distance effect without the necessity for direct contact with plants or pathogens. The aim of this study is to give an overview of the state-of-the-art in the field of *Bacillus*-based VOCs, especially in terms of their antibacterial, antifungal, and nematicidal action as the main segments determining their potential for biocontrol applications in sustainable agriculture.

## 1. Introduction

Microbial volatile organic compounds (mVOCs) represent a diverse group of molecules synthesized through different metabolic pathways by microorganisms, predominantly bacteria and fungi [1,2]. The role of mVOCs was explained through microorganisms’ evolution in the context of microbial communities due to their ability to mediate interactions with certain VOCs acting like infochemicals [1,3]. Bacteria use a cell-to-cell communication system (quorum sensing) as a method for monitoring the population density [3,4] relying on the synthesis, release, and subsequent detection of small diffusible signal molecules known as autoinducers [4]. Quorum sensing is variable depending on the bacteria type, allowing both intra- and inter-species communication. The chemical communication enabled by diffusible autoinducers is possible at short distances between cells, also requiring a high concentration of signaling molecules. Recent research, on the other hand, showed that microorganisms also produce different inorganic and organic volatile compounds that can be used as signals in intra- and inter-kingdom interactions, even at lower concentrations and over long distances [2]. This kind of communication system enables the coordinated behavior of a group of organisms to achieve process regulation leading to virulence, biofilm formation, and other developmental processes. The important finding in this matter was that microorganisms employ volatiles during interactions with plants, fungi, nematodes, and bacteria [4].

The beneficial effect of VOCs is additionally supported by several environmentally friendly properties, including biodegradability, meaning they do not leave toxic residues on plant surfaces, low molecular weight (100–500 Da), the lipophilic character with a low boiling point, and high vapor pressure (0.01 kPa), making them easily evaporative at normal temperature and pressure and enabling diffusion through the atmosphere and soil over different distances [2,5,6,7]. These characteristics enable them to fulfill their roles in plant growth and protection via pores in soil and rhizosphere environments. Additionally, mVOCs act as ideal signal/messenger molecules for mediating interactions at both short and long distances in microbes and plants [2,7]. One key characteristic suggested as the main difference compared to soluble compounds lies in the independence from solvents. Due to the restricted number of functional groups, many volatiles are defined as non-polar, showing low solubility in water that is sufficient to allow dissemination into the water phase. However, volatiles spread fast in both the gas and water phases. Through the gaseous phase, volatiles can spread in highly complex ecosystems such as soil, insect, and spider nests. This allows them to achieve functions such as communication and antimicrobial defense, which, on the other hand, cannot be performed by solvents due to the lack of effective spreading [8].

Based on chemical structure, mVOCs can be divided into different classes, including alkenes, alcohols, ketones, benzenoids, pyrazines, sulfides, and terpenes. The first systematic review on bacterial volatiles was published by Schulz and Dickschat in 2007 [9], describing in detail the biosynthesis of common volatiles such as fatty acids or sulfur compounds, which are predominantly produced by bacteria, as well as some not-so-common VOCs such as halogenated compounds. The research addressing the occurrence of volatiles in bacteria indicated that the compounds can be classified into three different groups: common, group, and specific compounds. Common compounds are being produced by different strains throughout the bacterial kingdom, and include basic fermentation products and primary metabolism-derived compounds. The typical representatives of the described group are dimethyl disulfide, 2-phenylethanol, aliphatic methyl ketones, and indole. The group compounds are mostly produced within certain genera or species. However, different mixtures made of the aforementioned compounds can be strain- or species-specific, resulting in specific biological effects [8,10,11]. The studies also confirmed that environmental parameters, including the medium composition in the first place but also temperature, humidity, pH, and oxygen levels, influence the composition of volatiles produced by microorganisms. The effects of the parameters can be direct, in terms of their physical properties (temperature and humidity will clearly affect molecule volatility), or indirect, by influencing the growth rate and metabolic activity. The presence or absence of oxygen has a strong impact on the production of volatile compounds, taking into account the mode of metabolism preferred by the particular producing strain, whether it is respiration or fermentation. Additionally, it is to be expected that volatiles’ production rate changes depending on the growth stage. Aside from abiotic factors, recent studies have shown that biotic factors, such as interactions between microorganisms of different species, can also cause or stop the release of volatiles [10]. This explains that volatiles are considered chemical signals that can be released specifically when needed and as results of complex two-way directed interactions existing between other members of the microbial community. Microorganisms can respond to a range of volatile compounds, which are diverse in terms of their structural and functional properties. However, a better understanding of how exactly cells perceive microbial volatiles is still limited and necessary due to the lack of information regarding the volatile-specific receptors in microorganisms. At this moment, it is confirmed that the physico-chemical properties of microbial volatiles make possible a passive diffusion through the cell wall or membrane. This implies that the target could be all cell components, including nucleoids, resulting in gene expression control. Taking into account the existing differences in volatile profiles among microorganisms, the relationship between them varies, and they are recognized within each other as friends, foes, or prey, consequently adjusting the behavior—persist, invade, escape, or defend. In microbial interactions, bacteria–bacteria, fungi–fungi, fungi–bacteria, or bacteria–protists relations, the response directly depends on the interacting partner; a volatile compound can have an opposite effect on different organisms [2,10,11].

The increased interest in microbial volatiles and a growing number of studies have been noticed in the last two decades, addressing their beneficial and environmentally friendly roles in the form of beneficial interactions among producing strains and plants. This results in the induction of systemic resistance against biotic and abiotic elements, plant growth promotion, and inhibition of plant fungal and bacterial pathogens [10,12]. The action mode of VOCs has an additional advantage over other biocontrol and growth-regulating mechanisms since the VOCs do not require any physical contact with pathogens or plant parts, in contrast with most of the other processes involved in controlling phytopathogens and promoting plant growth. In the scientific literature, it was reported that 300 bacteria and fungi are recognized as VOC producers. A total of 671 VOCs belong to 212 bacterial species, while 335 belong to 96 species of fungi, as recorded in the database of volatiles emitted by microorganisms [13]. The most common representatives also recognized based on beneficial activity that includes the influence on plant growth promotion and induction of systemic resistance are members of the *Bacillus, Pseudomonas, Stenotrophomonas, Serratia,* and *Arthrobacter* genus [12]. In recent years, the production of mVOCs has been considered a promising biocontrol method that enables short- and long-distance pathogen control [14]. The capability of volatiles to act as suppressive agents on neighboring pathogens and signal to plants confirms their potential role as sustainable alternatives to chemical fertilizers and pesticides [2]. The specificity of mVOCs in terms of target pathogens explains that the inhibitory effects of VOCs can mediate virulence factor expression of target pathogens, but also suppress metabolic processes, such as toxins biosynthesis [2,14]. Several recent studies are focused on a better understanding of possible modes of action in pathogens suppression, on the necessity to widen the investigation, and on addressing questions regarding clarifying the molecular targets of mVOCs across a broader range of pathogens. The studies also reported that the suppressing effects of mVOCs produced by bacterial mixtures in co-culturing methods can be significantly different from those produced by single species. In this kind of competitive interaction, mVOCs themselves can serve as signals to trigger or reduce the production of other mVOCs, with the additional possibility that some volatiles can be produced by precursors synthesized by different strains [7].

The potential of VOCs as biocontrol agents and eco-friendly alternatives to the chemical products conventionally used in agricultural practices for pest management is topic of a great scientific and practical interest recognized by many research groups worldwide. The present study is focused on an overview of the state-of-the-art in the field of *Bacillus*-based VOCs in terms of their antibacterial, antifungal, and nematicidal activity as the main factors determining their potential for biocontrol applications in sustainable agriculture. This study has summarized available scientific data regarding the Bacillus strains as VOC producers, focusing on clarifying the mechanisms of action and describing potential application routes, giving insights into the direction of the subsequent research steps and scientific questions addressed in the future.

## 2. Antibacterial *Bacillus*-Based VOCs and Their Mechanisms of Action

**Figure 1:**
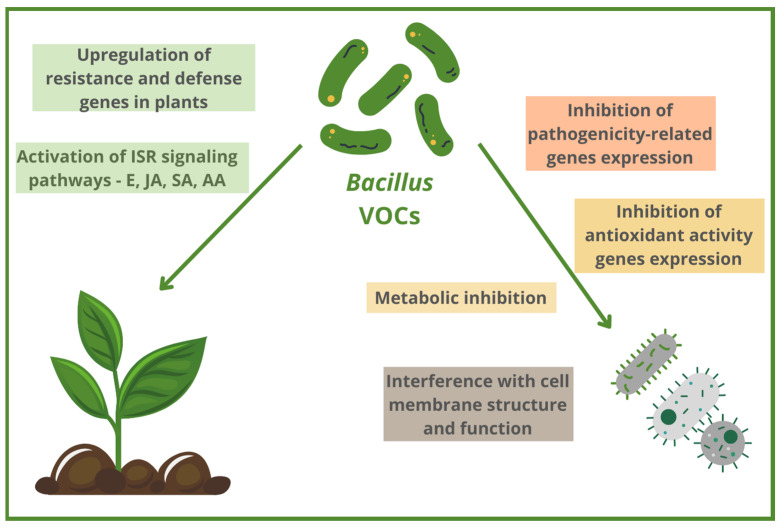
Mechanisms of antibacterial activity of *Bacillus*-based VOCs (ISR—induced systemic resistance; E—ethylene; JA—jasmonic acid; SA—salycilic acid; AA—abscisic acid).

**Figure 2:**
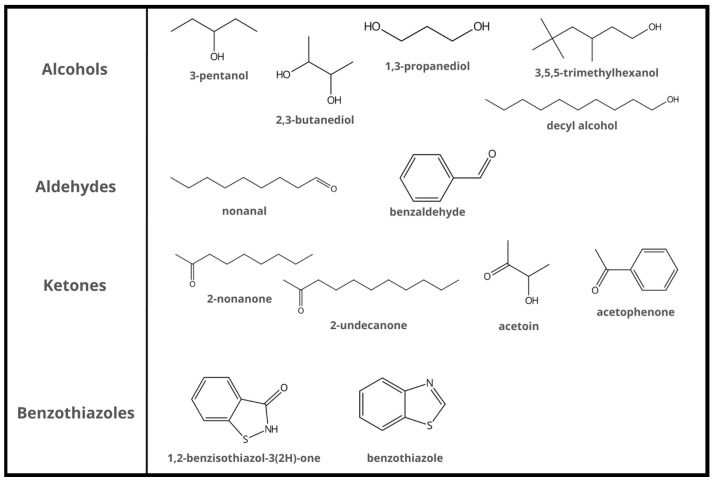
The most common antibacterial *Bacillus*-based VOCs.

Biocontrol activity of *Bacillus*-emitted VOCs against different plant pathogenic bacteria was previously reported in numerous studies (Table 1). Different mechanisms of antibacterial action against plant pathogens exhibited by the *Bacillus*-based VOCs are discussed below and summarized in Figure 1, while the most abundant antibacterial VOCs are presented in Figure 2.

### 2.1. ISR (Induced Systemic Resistance) Induction via Different Signaling Pathways as the Mechanism of Antibacterial Activity of Bacillus VOCs

Among the pioneers in this field, Ryu et al. [15] have proven the antibacterial effect of 2,3-butanediol produced by *Bacillus subtilis* GB03 and *B. amyloliquefaciens* IN937a against *Pectobacterium carotovorum* subsp. *carotovorum* (syn. *Erwinia carotovora* subsp. *carotovora*). The main mechanism of antibacterial action in the aforementioned study was ISR (induced systemic resistance) in *Arabidopsis thaliana* after 4 days of exposure to *Bacillus* VOCs, which was mediated through the ethylene-dependent signaling pathways, while the ISR was independent of the salicylic acid or jasmonic acid signaling pathways. VOCs produced by *B. subtilis* GB03 and *B. amyloliquefaciens* IN937a have shown a supreme biocontrol effect by reducing the number of symptomatic leaves to one in *Arabidopsis* plants, even in comparison to 2,3-butanediol applied as a pure compound (1–2 symptomatic leaves) as well as compared to water used as a control (4–5 symptomatic leaves) [15]. Acetoin (3-hydroxy-2-butanone) was found to be the main VOC produced by *B. subtilis* FB17 that triggers systemic resistance in *Arabidopsis thaliana* against *Pseudomonas syringae* pv. *tomato* DC3000 via induction of salicylic acid and ethylene signaling pathways, while the jasmonic acid signaling pathway was not essential for ISR. Plants treated with *B. subtilis* FB17 or acetoin as a pure compound have shown milder disease symptoms in terms of chlorosis, as well as a significant reduction of pathogen incidence measured as CFU/g of fresh weight (around 1 log unit) [16]. The antibacterial activity of 3-pentanol and 2-butanon supplied by the drench treatment in concentrations of 1 mM and 0.1 µM, respectively, was reported through the consistent triggering of systemic resistance in cucumber against *Pseudomonas syringae* pv. *lachrymans*, causing bacterial angular leaf spots, whereas 2,3-butanediol had been ineffective in eliciting induced resistance in cucumber plants in the aforementioned concentrations [17]. Later, it was reported that ten Petri dishes containing 10 µM 2,3-butanediol (the main VOC produced by *B. subtilis* GB03) were utilized in the miniature greenhouse system to successfully induce systemic resistance against *Pseudomonas syringae* pv. *lachrymans* on cucumber plants, which was mediated via the jasmonic acid signaling pathway. The disease severity score of cucumber angular leaf spot was reduced to 2.7 for *B. subtilis* GB03 VOCs treatment, compared to 4.2 for plants without VOCs treatment (on the scale in the range 0–5) [18]. The subsequent study revealed that 3-pentanol primes salicylic acid and jasmonic acid signaling pathways responsible for ISR towards *Pseudomonas syringae* pv. *tomato* in *Arabidopsis* plants [19]. Similar results regarding the ISR mechanisms were reported by Choi et al. [20], in which 3-pentanol from *B. amyloliquefaciens* IN937a has shown a significant reduction in pepper bacterial spots severity caused by *Xanthomonas axonopodis* pv. *vesicatoria* in field trials over two years. The disease index, measured 40 days after plant transplantation in the field, was reduced to 0.7 and 1.7 in 3-pentanol treatments, compared to 4.0 and 3.8 for water used as control (scale 0–5) in two subsequent years [20].

### 2.2. Modulation of Pathogens’ Gene Expression by Antibacterial Bacillus-Based VOCs

Inhibition of virulence traits and metabolic activity by inhibition of the related genes’ expression and modulating the metabolic pathways responsible for pathogenic behavior are among the most important mechanisms of action of *Bacillus*-generated VOCs in terms of their antibacterial activity. Furthermore, several studies have shown improved antibacterial activity achieved by the mixture of VOCs compared to separate compounds, thereby targeting the expression of different pathogenicity-related genes. The antibacterial activity of the VOC mixture produced by *B. amyloliquefaciens* SQR-9 was proven against *Ralstonia solanacearum* ZJ3721 (biovar 3), the causal agent of tomato bacterial wilt, with an efficiency of 70% in bacterial pathogen growth suppression when the VOC mixture was applied as a biocontrol agent, contrary to the 1–11% growth inhibition achieved by the individual VOCs [21]. The underlying mechanisms of antibacterial activity included inhibition of motility, biofilm formation, and root colonization by *Ralstonia solanacearum*, as well as inhibited production of antioxidant enzymes and exopolysaccharides, with significant down-regulation of genes involved in virulence traits, carbohydrate and amino acid metabolism, protein translation, and folding, as well as antioxidant activity [19]. Almost similar mechanisms of action were observed in the case of *B. amyloliquefaciens* T-5 VOCs antibacterial activity against the tomato bacterial wilt pathogen, where pathogen growth inhibition rates of 75%, 62%, and 85% were achieved using the mixture of VOCs produced by the *Bacillus* antagonist on the agar medium, in sterilized soil, and natural soil, respectively [22]. This study also reported down-regulation of several virulence- and metabolism-related genes of *Ralstonia solanacearum* in the range 27–54%, including transcriptional regulator (*phcA*), catalase (*katG*), and SOD (superoxide dismutase—*sobB*) genes motility-related genes (twitching motility (*pilT*) and flagellin (*fliC*)) as well as metabolism-related genes, DNA polymerase (*polA*) and pyruvate dehydrogenase (*aceE*) [22]. The same research group has also investigated induced VOC production in the presence of organic fertilizers prepared from different animal and plant organic waste materials [23], where the production of VOCs was significantly improved when combined with the application of organic fertilizers. This suggests a possible novel good agricultural practice in tomato bacterial wilt management. It was concluded that maximal production of 2-nonanone, nonanal, xylene, benzothiazole, and butylated hydroxy toluene was achieved by the strain *B. amyloliquefaciens* T-5 in the presence of organic fertilizer made of amino acid organic fertilizer (prepared from the microbially hydrolyzed oil rapeseed cake) and pig manure compost (1:1, *w*/*w*). On the other hand, maximal production of 2-nonanone, nonanal, xylene, and 2-undecanone was achieved by *B. amyloliquefaciens* SQR-9 in the presence of organic fertilizer composed of 41% vinegar-production residue, 20% rice straw, and 39% cattle dung [23].

The suppression of *Ralstonia solanacearum* as the causal agent of tobacco bacterial wilt was also investigated by Tahir et al. [12,24]. Albuterol and 1,3-propanediol produced by *B. subtilis* SYST2 were found to be responsible for ISR and growth promotion of tobacco plants at the level of inhibition of gene expression related to ethylene production, while on the other hand, genes related to expansin, wilt resistance, and plant defense were overexpressed. Moreover, the wilt index in tobacco plants was reduced from 90.66% to 33.00%, 19%, and 27% by applying inoculation with *B. subtilis* SYST2 or treatments with 1,3-propanediol (1 mM) and albuterol (0.1 mM) [12]. Benzaldehyde, 1,2-benzisothiazol-3(2H)-one, and 1,3-butadiene were found to be major VOCs produced by *B. amyloliquefaciens* FZB42 and *B. atrophaeus* LSSC22, which suppressed *Ralstonia solanacearum* in tobacco plants by reducing the colony size, pathogen cell viability, and motility, and negatively influencing chemotaxis. One of the major mechanisms of action was the induction of morphological and ultrastructural changes/abnormalities in *Ralstonia solanacearum* cells. Furthermore, expression of pathogenicity-related genes was significantly down-regulated, including *PhcA* (a global virulence regulator), type III secretion system (T3SS), type IV secretion system (T4SS), extracellular polysaccharides, and chemotaxis-related genes. On the other hand, genes related to pathogen resistance and defense were significantly overexpressed, where the salicylic acid pathway was found to be responsible for the induction of systemic resistance, with up-regulation of the *EDS1* and *NPR1* genes as its main components. All of the aforementioned mechanisms of action have resulted in the reduced tobacco wilt index during *in planta* experiments (28% for *B. amyloliquefaciens* FZB42 and 43.2% for *B. atrophaeus* LSSC22 VOCs compared to 98% for the non-exposed control) [24].

Growth inhibition of *Ralstonia solanacearum* was also achieved by 3,5,5-trimethylhexanol produced by *B. cereus* D13, which has also been successful in suppression of *Pseudomonas syringae* pv. *tomato* DC3000 and *Xanthomonas oryzae* pv. *Oryzicola*. This is while both 3,5,5-trimethylhexanol and decyl alcohol have shown antibacterial activity against *Xanthomonas oryzae* pv. *oryzae* with growth inhibition of 60.7% and 53.6% at minimum inhibitory amounts of 0.48 and 2.4 mg, respectively [25]. The same study has revealed several mechanisms of antibacterial activity against *Xanthomonas oryzae* pv. *oryzae* as the causal agent of bacterial leaf blight in rice. The first one was the inhibition of pathogen swimming and swarming motility after 3-day exposure to *B. cereus* D13 VOCs, with significant down-regulation of the genes *motA*, encoding flagellar motor component MotA, and *motC*, encoding the flagellar motor protein. Interestingly, while pathogen motility was restricted during the first 3 days of exposure, it took 6 days of exposure to observe a sharp decline in the number of viable pathogen cells, indicating that a certain period of time was required to achieve lethal VOCs concentration, while a certain extent of pathogen spreading inhibition could be achieved in earlier phases of antagonist-pathogen interaction at the sub-lethal level of VOCs concentration. Considering that the rhizosphere is a relatively closed environment, it is a favorable place for maximization of VOCs’ antibacterial activity, considering the shorter time necessary to achieve a certain VOCs concentration threshold required for bactericidal effect. In the case of *Xanthomonas oryzae* pv. *oryzae*, rice rhizosphere is therefore a good place to apply antagonist or VOCs solely in order to prevent or inhibit infection via root wounds [25].

### 2.3. Structural and Functional Changes at Cell Level Caused by Antibacterial Bacillus VOCs

Another mechanism of antibacterial activity was observed in the case of *B. cereus* D13 VOCs, including alteration of *Xanthomonas oryzae* pv. *oryzae* cell membrane permeability resulting in changed cell morphology, which could be directed in two ways: (a) increased membrane permeability through membrane distortion, resulting in leakage of intracellular contents, and (b) decreased membrane permeability resulting in concentrated cytoplasm [25]. Hence, the action mode of 3,5,5-trimethylhexanol and decyl alcohol VOCs could be explained by their ability to cause leakage or impede the interchange of materials at the cell membrane surface [4].

On the other hand, one of the possible antibacterial activity mechanisms related to pathogen cells’ morphological changes is also inducing several cell abnormalities at the ultrastructural level, as mentioned before [24]. The same was observed by Rajer et al. [5], where benzaldehyde, nonanal, benzothiozole, and acetophenone emitted by *B. subtilis* FA26 induced a wide range of abnormalities in cells of *Clavibacter michiganensis* ssp. *sepedonicus*, a causal agent of potato bacterial ring rot, including distorted colony morphology, misshapen cells and their disintegration, the formation of inclusions, movement of cytoplasmic content toward the ruptured cytoplasmic membrane, and lack of cytoplasmic content or fragmented cytoplasm due to intracellular content leakage. The same authors concluded that the production and activity of *B. subtilis* FA26 VOCs depend on cultivation conditions and that an increase in their biocontrol potential against *Clavibacter michiganensis* ssp. *sepedonicus* is related to the increased VOC concentrations [5].

### 2.4. Effects of Antibacterial Bacillus VOCs Concentration and Treatment Timing on Biocontrol Efficiency

Besides cultivation conditions, the other important variable determining the efficacy of the VOC treatment is the time of application, considering the current pathogen growth and development stage. Han et al. [25] have shown that the mixture of VOCs produced by *B. velezensis* strains JCK-1618 and JCK-1696 was more successful in suppressing *Xanthomonas arboricola* pv. *pruni* when *B. velezensis* strains were inoculated three days before pathogen inoculation, compared to a one-day window between the antagonist and pathogen inoculation, where the pathogen suppression efficacy ranged between 22.4% and 72.2%.

A mixture of *B. velezensis* X5-2, *B. megaterium* X6-3, and *Pseudomonas orientalis* X2-1P VOCs, including alkanes, alkenes, pyrazines, acids, alcohols, and indoles, was found to be effective during the in vitro and in vivo suppression of the winter oilseed rape black rot pathogen *Xanthomonas campestris* pv. *campestris*, with disease reductions of 82.37% and 72.47% in preventive and curative treatments, respectively [26]. Pyrazines (pyrazine, 2-ethyl-3-methyl, pyrazine, 2-ethyl-, pyrazine, 2, 5-dimethyl, and pyrazine, 2-methyl) were also found as active VOCs of *B. megaterium* BmBP17 against the bacterial wilt pathogen *Ralstonia solanacearum*, where pyrazine, 2-ethyl-3-methyl, was found to be the most effective compound with a required dosage of 672 μg/mL for complete inhibition of *Ralstonia solanacearum* [27]. This study also confirmed an increase in antibacterial activity with an increase in VOC concentrations.

**Table 1:** Literature overview concerning VOCs exhibiting antibacterial activity against plant pathogens produced by *Bacillus* spp.

*Bacillus* Strain	Plant Pathogen (Disease)	Antibacterial VOCs	Reference
*B. subtilis* FB17	*Pseudomonas syringae* pv. *tomato*	acetoin	Rudrappa et al. [16]
*B. subtilis* FA26	*Clavibacter michiganensis* ssp. *sepedonicus* (potato bacterial ring rot)	benzaldehyde nonanal benzothiazole acetophenone	Rajer et al. [5]
*B.s subtilis* SYST2	*Ralstonia solanacearum* (tobacco bacterial wilt)	albuterol 1,3-propanediol	Tahir et al. [12]
*B. subtilis* GB03	*Pseudomonas syringae* pv. *lachrymans*	2,3-butanediol	Song et al. [18]
*B. amyloliquefaciens* IN937a	*Xanthomonas axonopodis* pv. *vesicatoria* (pepper bacterial spot)	3-pentanol	Choi et al. [20]
*B. amyloliquefaciens* T-5	*Ralstonia solanacearum* (tomato bacterial wilt)	mixture of VOCs	Raza et al. [22]
*B. amyloliquefaciens* SQR-9	*Ralstonia solanacearum* (tomato bacterial wilt)	mixture of VOCs	Raza et al. [21]
		2-nonanone 2-undecanone nonanal xylene benzothiazole butylated hydroxy toluene	Raza et al. [23]
*B. megaterium* BmBP17	*Ralstonia solanacearum*	pyrazine, 2-ethyl-3-methyl pyrazine, 2-ethyl- pyrazine, 2, 5-dimethyl pyrazine, 2-methyl	Munjal et al. [27]
*B. cereus* D13	*Xanthomonas oryzae pv. oryzae* (rice bacterial leaf blight) *Xanthomonas oryzae pv. oryzicola* *Pseudomonas syringae pv. tomato* *Ralstonia solanacearum*	decyl alcohol 3,5,5-trimethylhexanol	Xie et al. [25] Xie et al. [4]
*B. atrophaeus* JZB120050	*Ralstonia solanacearum* *Pseudomonas tolaasii* *Pseudomonas syringae pv. lachrymans*	mixture of VOCs	Ni et al. [28]
*B. subtilis* GB03 *B. amyloliquefaciens* IN937a	*Erwinia carotovora* subsp. *carotovora*	2,3-butanediol acetoin	Ryu et al. [15]
*B. amyloliquefaciens* FZB42 *B. atrophaeus* LSSC22	*Ralstonia solanacearum* (tobacco bacterial wilt)	benzaldehyde 1,2-benzisothiazol-3(2H)-one 1,3-butadiene	Tahir et al. [24]
*B. velezensis* X5-2 *B. megaterium* X6-3 *Pseudomonas orientalis* X2-1P	*Xanthomonas campestris* pv. *campestris* (black rot of winter oilseed rape)	mixture of VOCs	Jelušić et al. [26]
*B. velezensis* JCK-1618 *B. velezensis* JCK-1696	*Xanthomonas arboricola* pv. *pruni*	mixture of VOCs	Han et al. [25]

## 3. Mechanisms of Action of Antifungal *Bacillus*-Based VOCs

**Figure 3:**
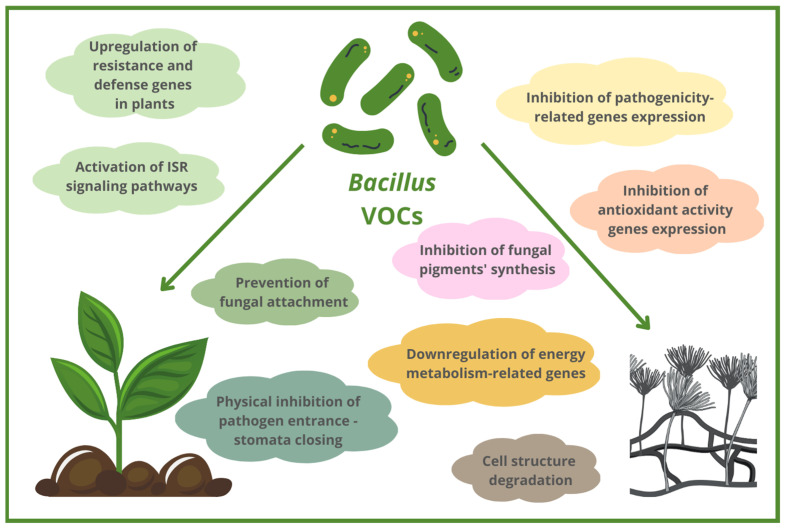
Antifungal mechanisms of action of *Bacillus*-based VOCs (ISR—induced systemic resistance).

**Figure 4:**
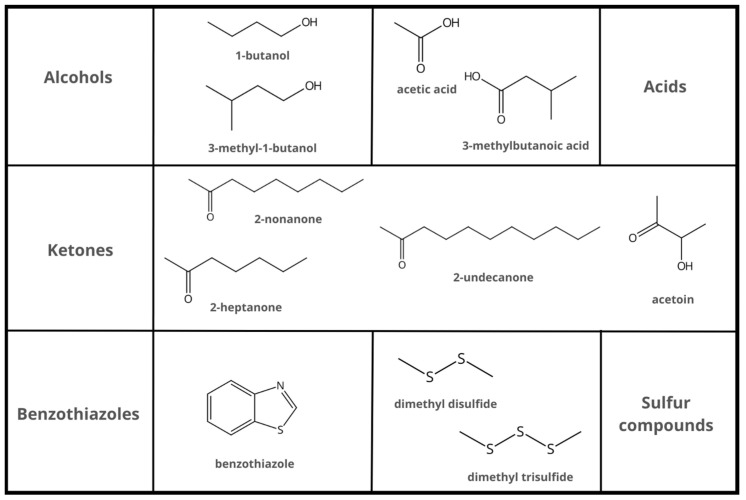
The most common antifungal VOCs produced by *Bacillus* spp.

The overview of published literature data investigating the antifungal activity of *Bacillus*-based VOCs shows a supreme number of studies compared to literature investigating antibacterial and nematicidal effects. Hence, a general overview of the mechanisms involved in the suppression of plant-pathogenic fungi by the *Bacillus* VOCs will be given here (summarized in Figure 3), emphasizing the most abundant and effective VOCs in terms of antifungal activity. A more detailed overview of the published data regarding the antifungal activity of VOCs produced by different strains of the genus *Bacillus* can be found in Table 2, while the most abundant *Bacillus*-based VOCs with antifungal activity towards plant pathogens are presented in Figure 4.

### 3.1. Cultivation and Treatment Variables Affecting Antifungal Efficiency of Bacillus VOCs

The effect of the growth medium on VOCs production and antibacterial activity was reported by Huang et al. [29], where 90% growth inhibition of *Rhizoctonia solani* and *Pythium aphanidermatum* (Edson) was achieved using *B. mycoides* grown on TSA (tryptic soy agar) or SPMA (soy powder milk agar). This is while the application of KBA (King’s B agar) and LBA (Luria-Bertani agar) resulted in moderate pathogen growth inhibition and NA (nutrient agar) and PDA (potato dextrose agar) showed almost no inhibitory activity against fungal pathogens. Similar results in terms of the suitability of TSA as a growth medium were obtained by Gotor-Vila et al. [30] for *B. amyloliquefaciens* CPA-8, whose antifungal activity was the highest compared to NYDA (nutrient yeast glucose agar) and NAglu20 (nutrient agar supplemented with glucose) in the cases of *Monilinia laxa* (>78.6%), *Botrytis cinerea* (86.8%), and *Monilinia fructicola* (68.6%) suppression. In the study by Fujimoto et al. [31], TSB (tryptone soya broth) and TSA favored the production of VOCs by *Bacillus* sp. ACB-65 and *Bacillus* sp. ACB-73, which exhibited antifungal activity against *Phyllosticta citricarpa*, the orange-black spot pathogen, over the NA, PDA, and King B media. Specific VOCs were produced by *B. amyloliquefaciens* strains UCMB5033, UCMB5036, UCMB5113, and FZB42 using different cultivation media: 2,3-butanedione and acetoin on M9A medium, while 5-methyl-heptanone, 2-methylpyridine, and 2-pentanone were produced on TSA and LBA media [32]. The highest production of antifungal VOCs (chloroacetic acid, tetradecyl ester, octadecane and hexadecanoic acid, and methyl ester) by *B. atrophaeus* HAB-5 in submerged cultivation was obtained in LB medium, followed by BPY (beef peptone yeast) and MH (Mueller Hinton) medium, inhibiting the growth of *Colletotrichum gloeosporioides* by 47.81, 45.51, and 44.55%, respectively [33]. Different types of growth media (NA, TSA, LBA, and TMEA (TM Enterprise agar) were investigated for their effect on VOCs production by *B. pumilus* TM-R and their antifungal activity against *Alternaria alternata, Aspergillus niger, Cladosporium cladosporioides, Curvularia lunata, Fusarium oxysporum*, *and Penicillium italicum* in both small- and large-scale tests (plate and 12L-tests, respectively). TMEA medium resulted in the strongest antifungal activity, supported by the production of methyl isobutyl ketone, ethanol, 5-methyl-2-heptanone, and S-()-2-methylbutylamine as the predominant antifungal VOCs [34]. MOLP medium (medium for optimum lipopetide production) used for the production of VOCs by *B.velezensis* BUZ-14 and *B. ginsengihumi* S38 resulted in 90% suppression of *Botrytis cinerea* in table grapes, while grape juice was the least favorable medium for VOC efficacy [35].

Furthermore, cultivation time also affects the type and content of the bacterial VOCs produced. The largest diversity of VOCs in the cultivation broth of *B. subtilis* CF-3 was detected after 48 hof cultivation, while the peak of the antifungal efficiency (73.46% on *Monilinia fructicola*, causing peach brown rot, and 63.63% on *Colletotrichum gloeosporioides*, causing litchi antrachnose), was achieved after 24 h of cultivation. Benzothiazole and 2,4-di-tert-butylphenol showed a strong inhibitory effect on both pathogens in vitro and vivo [36], with EC_50_ values of 9.90 × 10^−4^ mol/L for *Monilinia fructicola* and 1.26 × 10^−2^ mol/L for *Colletotrichum gloeosporioides* [37]. Similar results were obtained by Ni et al. [28], where *B. atrophaeus* JZB120050 produced 29 alkanes, three alkenes, four acids, one aldehyde, and one phenol after 24 h, while 41 compounds were detected via GC-MS analysis, including 34 alkanes, three alkenes, two acids, one benzene, and one phenol after 48 h of cultivation. Zheng et al. [38] investigated the mixture content of VOCs produced by *B. amyloliquefaciens* PP19 to suppress *Peronophythora litchi* and found significant differences in VOCs production over the course of the cultivation, where a total of 9, 33, 14, 28, and 17 compounds were detected at each of the five investigated time points (24, 36, 48, 60 and 72 h, respectively). However, only two compounds (2-nonanone and 6-methyl-2-heptanone) were common at all time points and constantly produced during the cultivation. Zhang et al. [39] have found that there was no significant difference in biocontrol activity regarding the in vivo suppression of raspberry postharvest diseases caused by *Botrytis cinerea* and *Rhizopus stolonifer* when VOCs produced by *B. siamensis* G-3 in NA medium and at pH 7.0 were applied for treatment after 72 h and 84 h of cultivation. This points out the necessity to optimize cultivation time in order to achieve maximal VOCs biocontrol efficacy while minimizing production operational costs.

The timing of inoculation/VOCs in correspondence with the pathogen infection time frame has been considered an important aspect affecting the efficiency of plant pathogen suppression, pointing out the importance of choosing between the preventive/curative plant disease management strategies. Previously mentioned molecular signaling between antagonists and plant pathogens and the related induction of VOC production in the pathogen’s presence need to be understood in more detail to be able to define the appropriate timing of the treatment to maximize its efficiency. For example, Han et al. [25] have shown that the inoculation of antagonists *B. velezensis* JCK-1618 and JCK-1696 3 days before the fungal pathogen inoculation has significantly increased the treatment efficiency compared to a shorter time period (1 day) between the subsequent antagonist and pathogen inoculation. The efficiency of the VOCs produced by *B. velezensis* JCK-1696 was increased from 67% to 75.7% inhibition for *Mycosphaerella cerasella* and from 44.9% to 66.8% inhibition for *Epicoccum tobaicum*, taking into account a 1-day and 3-day inoculation interval [25]. These findings support several mechanisms of action described for *Bacillus* spp. biocontrol activity, including competition for growth space and nutrients with extra time given to establish antagonist populations at the target site of application as well as the longer time necessary to achieve a fungicidal level of VOC content. Jangir et al. [40] have reported an increase in the antifungal activity of *Bacillus* sp. B44 VOCs against *Fusarium oxysporum* f. sp. *lycopersici* from 20% on the 1st day of the in vitro experiment to 70% on the 7th day of incubation, while the in vivo test showed a 36% reduction in tomato disease incidence. An increase in the antifungal activity of VOCs produced by *B. amyloliquefaciens* ALB629 and UFLA285—including 3-methylbutanoic acid, 2-methylbutanoic acid, isovaleric acid, and 2- methyl butyric acid—was observed in the reduction of common bean antrachnose from 83% on the first day to 93% disease incidence reduction on the 11th day [41]. This was also shown by Arrebola et al. [42], where VOCs produced by *B. amyloliquefaciens* PPCB004 exhibited the highest inhibition of fungal radial growth of *Penicillium crustosum* (73.3%) in vitro after 10 days. This is while a significant in vivo decrease of decay incidence and severity in Valencia orange was observed after a 12-day treatment. Similar results were observed by Leelasuphakul et al. [43], where the disease incidence of green mold caused by *Penicillium digitatum* Sacc. in citrus fruit was decreased by 86.7% and disease symptoms were delayed by 6 days and decay symptoms to day 9 by the VOCs produced by the *B. subtilis* 155 suspensions, which was inoculated 24 h before pathogen inoculation. *B. amyloliquefaciens* NJN-6 inhibited the mycelial growth of *Fusarium oxysporum* f. sp. *cubense* by 30% to 40% compared with the control after 3 days of treatment, while in the soil test the number of *Fusarium oxysporum* f. sp. *cubense* was significantly reduced (10^2^ spores/g compared to 10^4^ spores/g in control samples) after 45 days of treatment [44]. The same study identified benzene (2,3,6-trimethyl-phenol) and ketone (2-undecanone, 2-dodecanon, and 2-tridecanone) compounds as the most effective in terms of antifungal activity, where the number of carbon atoms in ketones negatively correlated with their antifungal activity against *Fusarium oxysporum* f. sp. *cubense* [44]. *B. subtilis* Bs 8B-1 has produced a sufficient amount of antifungal VOCs after 5 days of incubation with *Phytophthora capsici*, causing cucumber damping-off, and *Rhizoctonia solani*, causing radish damping-off [45]. *B. velezensis* RDA1 VOCs inhibited the growth of *Rosellinia necatrix* by approximately 60–70% compared to control after 10 days of treatment [46].

### 3.2. Wide Spectrum of Bacillus VOCs Antifungal Activity

A wide spectrum of antifungal activity for the mixture of *Bacillus*-derived VOCs as well as for separate VOCs has been reported widely in the literature. For example, the soil isolate *B. subtilis* G_8_ has inhibited mycelial growth and completely prevented the pigment production of different soil-borne pathogens, where inhibition rates of *Sclerotinia sclerotiorum, Botrytis cinerea,* and *Cercospora kikuchii* Chupp were over 75%, while inhibition rates were less than 46% for *Alternaria brassicae* and *Rhizoctonia solani* [47]. DG4 (an isomer of acetylbutanediol) produced by *B. subtilis* C9 has shown strong antifungal activity in terms of inhibition of mycelial growth against a wide range of fungal pathogens, including *Colletotrichum gloeosporioides* (44.41%), *Chatomium globosum* (59.38%), *Glomerella cingulata* (73.40%), *Fusarium oxysporum* (93.61%), *Aspergillus niger* (78.97%), *Corynespora cassiicola* (44.37%) and *Rhizoctonia solani* (79.14%) [48]. Chaves-Lopez et al. [1] have ranked several fungal strains according to their sensitivity to the VOCs produced by *B. subtilis, B. amyloliquefaciens*, and *B. cereus* as follows: *Moniliophthora perniciosa* > *Aspergillus niger* > *Fusarium oxysporum* f.sp. *lactucae* and *Aspergillus flavus* > *Aspergillus clavatus* > *Aspergillus parasiticus*. For example, 1-butanol, 3-methyl-1-butanol, acetic acid, 2-methylpropanoic acid, carbon disulphide, 3-methylbutanoic acid, and ethyl acetate successfully inhibited the mycelial growth of *Moniliophthora perniciosa* by about 84% and that of *Fusarium oxysporum* f.sp. *lactucae* by about 50% [1]. *B. megaterium* USB2103 VOCs have also been shown to have antifungal activity against a wide range of fungi, including *Botrytis cinerea, Phytophthora nicotianae, Rhizoctonia solani, Sclerotinia sclerotiorum, Verticillium dahliae*, as well as *Fusarium oxysporum* and *Macrophomina phaseolina*, with acetic acid and 2-nonanone as dominant antifungal VOCs [49]. *B. velezensis* ZSY-1 isolated from Chinese cabbage has produced VOCs exhibiting significant antifungal activity against *Alternaria solani* (81.1%), *Botrytis cinerea* (93.8%), *Valsa mali* (83.2%), *Monilinia fructicola* (80.9%), *Fusarium oxysporum* f. sp. *capsicum* (76.7%), and *Colletotrichum lindemuthianum* (70.6%). Among the produced VOCs, pyrazine (2,5-dimethyl), benzothiazole, 4-chloro-3-methyl, and phenol-2,4-bis(1,1-dimethylethyl) had shown significant antifungal activity against *Alternaria solani* (87.5%, 100%, 100%, and 89.14%, respectively) and *Botrytis cinerea* (100%, 100%, 100%, and 91.19%, respectively) [50]. *B. amyloliquefaciens* DA12 VOCs inhibited the growth of six pathogens including *Botrytis cinerea* (cucumber grey mold), *Colletotrichum coccodes* (pepper anthracnose), *Endothia parasitica* (chestnut blight), *F. oxysporum* f. sp. *lycopersici* (tomato wilt), *Raffaelea quercus-mongolicae* (oak wilt), and *Rhizoctonia solani* (rice sheath blight), with inhibition rate in the range 31.6–95.5%, as well as mycelial growth and mycotoxin production by eight toxigenic *Fusarium* spp. (*Fusarium asiaticum, Fusarium graminearum, Fusarium proliferatum, Fusarium verticillioides,* and *Fusarium oxysporum)* producing nivalenol, deoxynivalenol (and its acetylated derivatives), and fumonisin, with an inhibition rate ranging from 57.1% to 74.0% [51]. Inhibition of mycotoxigenic fungi was also observed by the VOCs produced by *B. megaterium* KU143 and *Pseudomonas protegens* AS15 in the case of *Aspergillus flavus*, where the total aflatoxin production in rice grains was reduced by 90.4%, besides the reduced fungal population [52]. Hlebová et al. [53] have shown complete inhibition of ochratoxin synthesis in *Aspergillus ochraceus* and *Aspergillus westerdijkiae* by VOCs produced by *B. mycoides, B. subtilis,* and *B. thuringiensis*. Acetoin was found to be the major VOC produced by *B. velezensis* G341, inhibiting mycelial growth of *Sclerotinia sclerotiorum* (cucumber *Sclerotinia rot*), *Rhizoctonia solani* (rice sheath blight), and *Botrytis cinerea* (tomato grey mold) [54]. VOCs in a 24-h cultivation broth of *B. subtilis* CF-3 inhibited the mycelial growth of *Botrytis cinerea, Colletotrichum gloeosporioides, Penicillium expansum, Monilinia fructicola*, and *Alternaria alternata,* with a mean inhibition rate of 59.97% [35]. Biovolatiles produced by *B. velezensis* NKG-2 suppressed the mycelial growth of *Fusarium oxysporum* (57%), *Fusarium graminearum* (68%), *Botrytis cinerea* (57%), *Alternaria alternata* (46%), *Fulvia fulva* (65%), and *Ustilaginoidea virens* (51%) [55].

### 3.3. Morphological and Ultrastructural Abnormalities in Fungal Cells Caused by Bacillus VOCs

Mycelia morphological abnormalities were observed in *Sclerotinia sclerotiorum* by Liu et al. [56], together with spore cracking causing the brownish color of sporaceous inclusion and its effusion after 24–48 h of treatment using *Paenibacillus polymyxa* BMP-11, *B. subtilis* BL02, *B. pumilus* BSH-4, and *B. pumilus* ZB13 VOCs. Several VOCs from *B. megaterium* USB2103 have caused ultrastructural alterations at cell organelles, mostly membranes, mitochondria, and endoplasmic reticulum of *Sclerotinia sclerotiorum* [49]. Hemolytic activity of the majority of the pure VOCs confirmed that the membrane appeared to be one of the primary targets in terms of the antifungal mechanism of action since 2-nonanone caused damage to the cytoplasmic membrane, resulting in complete or partial hyphae emptying. Detachment of the membrane from the outer cell wall resulted in strong vacuolization with internal residues of membranes in the cytoplasm. DL-limonene treatment has also led to cytoplasmic membrane detachment from the cell wall, as well as cytoplasm granulation, the absence of organelles, multi-vesciculation, and the accumulation of protein and lipidic material in the cytoplasm. Dimethyl disulfide has shown strong ultrastructural modifications, including missing or altered cytoplasm, hyper-vesiculation, hypocrested and vesiculated mitochondria, and accumulation of protein and lipidic material in the cytoplasm [49]. Ultrastructural damage of *Sclerotinia sclerotiorum* hyphae due to the VOCs of *B. velezensis* VM11 included abnormalities on cell membranes, mitochondria, nucleus, multivesicular structures, and cytoplasm, together with increased vacuoles’ size and disorganized cytoplasmic materials to the extent of non-descript cell organelles [57]. On the other hand, Monteiro et al. [58] have not observed any hyphae alteration in *Sclerotinia sclerotiorum* causing white mold in *Lactuca sativa* after contact with VOCs produced by *B. subtilis*, although the reduction of mycelial growth was 83.84%, indicating the fungistatic effect of the produced VOCs. 

Abnormalities on conidiophores of *Penicillium crustosum* were observed in treatment with VOCs produced by *B. subtilis* PPCB001, while complete loss of conidiophore structures was detected in combined treatment with *B. amyloliquefaciens* PPCB004 VOCs, with the presumed dominant role of 3-hydroxy-2-butanone (acetoin), which has induced reduction of the multiple phialides at the end of each hyphae, as well as vacuolation and swelling in hyphae and sporangium [41]. Besides the reduced radial growth in vitro, dimethyl disulphide and ammonia as the main VOCs produced by *B. mycoides* had reduced the damping-off disease of cabbage seedlings caused by *Pythium aphanidermatum* Edson by 45% in greenhouse experiments, while the disease incidence caused by *Rhizoctonia solani* had not been reduced. The bacterial VOCs affected the morphology of fungal hyphae, causing poor rigidity, shrinkage, curling, swelling, and hyphal deformation, together with organelle degeneration [29]. Abnormal swelling and increased branching of the *Fusarium oxysporum* f. sp. *niveum* hyphae were induced by VOCs produced by *B. subtilis* IBFCBF-4 [59]. 

*O*-anisaldehyde produced by *B. atrophaeus* CAB-1 showed a higher in vitro inhibitory effect than L-alaninol on hyphal elongation of *Botrytis cinerea* when applied at the concentration of 80 µL/plate (70.2% growth inhibition). Interestingly, the individual VOCs produced by *B. atrophaeus* CAB-1 have not been successful in the biocontrol of cucumber powdery mildew (*Sphaerotheca fuliginea*), while the mixture of VOCs has shown supreme biocontrol efficacy (71.54%) under greenhouse conditions compared to the other bioactive compounds of the same strain, including lipopeptides (29.57%) and crude secreted proteins (41.94%) [60]. Several VOCs produced by *B. subtilis* M29, including 2,6-diisocyanato-1-methyl-benzene, 1-propoxy-2-propanol, and benzophenone, were reported to destroy normal hyphae morphology and induce mycelial fragmentation and crumpling in *Botrytis cinerea* [61]. *Botrytis cinerea* hyphae treated with *B. velezensis* XT1 and *B. atrophaeus* L193 volatiles produced in the MOLP medium showed severe cytoplasmic cavitation and vacuolation, and no organelles were identified, while the reduction of fungal growth was 27% and 46%, respectively [62].

Chaves-Lopez et al. [1] have reported changes in colony morphology, spore production, and microstructural changes of the hyphae in different fungal pathogens induced by the VOCs produced by *B. subtilis, B. amyloliquefaciens,* and *B. cereus*. The presence of VOCs from *B. subtilis* SV75-1 has significantly contributed to degenerative changes in the hyphal morphology of *Moniliophthora perniciosa*, including the observation of flaccid hyphae presenting retracted protoplasm and the formation of empty segments and a thinner wall. Retraction of protoplasm was also observed in *Aspergillus parasiticus* exposed to VOCs from *B. cereus* SV40, while exposure to *B. amyloliquefaciens* SV20-2 VOCs resulted in shortened and swollen somatic hyphae. *Fusarium oxysporum* f. sp. *lactucae* MA284 exposure to the VOCs of *B. subtilis* SV75-1 has resulted in a reduction in conidia number and granulation of the mycelia [1].

Malformations, vacuolations, and swellings were observed in hyphae of *Verticillium dahliae*, causing tomato *Verticillium* wilt as induced by *B. velezensis* C2 VOCs and resulting in an approximately 70% reduction in disease incidence [63]. The hyphae of *Verticillium dahliae* were completely lysed/dissolved after a 5-day treatment with styrene produced by *Bacillus* sp. T6 [64].

The VOCs produced by *B. vallismortis* 12a and *B. altitudinis* 14b seriously decomposed the cell walls and damaged the protoplast of *Monilinia fructicola*, causing peach brown rot, with a special emphasis on the outer cell walls, whose structure was thin or gapped, which might allow leaking out of the cell contents. Additionally, the result was 77.1% and 50% disease suppression in fumigation treatment with cultivation broth of these two strains, respectively [65]. Similar thin or gapped structures of the uneven cell wall surface presenting a retracted protoplasm were also observed in *B. cereus* CF4-51 VOCs-treated mycelia of *Sclerotinia sclerotiorum* [66]. Acetic acid (20.68%), propanoic acid (33.30%), butanoic acid (26.87%), valeric acid (43.71%), and isovaleric acid (53.10%) produced by *Bacillus* sp. LPPC170 significantly inhibited the mycelial growth of *Fusarium kalimantanense*, causing Panama disease of banana, by damaging the vegetative and reproductive structures of the fungus and causing a lower density of mycelium with dehydrated, considerably deformed, withered, and malnourished hyphae [67].

Hyphae with wrinkled surface cells, surface swelling, and thinner cell walls were observed as a consequence of 6-methyl-2-heptanone (emitted by *B. subtilis* ZD01) treatment in *Alternaria solani*. As a result, the cytoplasm was shrunken, with an increased number of inclusions and larger liquid droplets, together with cytoplasmic content movement toward the ruptured cell walls or cytoplasmic membranes [68]. Hyphae of *Alternaria alternata* (tobacco brown spot) without any attached conidia appeared ruptured, shrunken, and twisted after being treated with 2-methylbutanoic acid and 3-methylbutanoic acid produced by *B. siamensis* LZ88 [69]. The mycelium of *Alternaria solani*, the causal agent of tomato early blight, became thin, twisted, deformed, bifurcated, and fractured, with a wrinkled and cracked surface, followed by intracellular content leakage after the treatment with *B. velezensis* ZJ1 VOCs led by isooctanol and 2-nonanol as the major active compounds [70]. Similar results were found for *Alternaria solani* causing early potato blight when exposed to the VOCs produced by *B. subtilis* ZD01 [71], as well as for *Mucor circinelloides*, *Fusarium arcuatisporum, Alternaria iridiaustralis,* and *Colletotrichum fioriniae* due to 2,3-butanedione and 3-methylbutyric acid produced by *B. subtilis* CL2 [72]. Twisted, flattened, and enlarged hyphae that lost their linearity were observed in *Alternaria iridiaustralis*, caused by the VOCs produced by *B. velezensis* L1 with 2,3-butanedione as the leading VOC [73].

Twisted hyphae were also observed in Colletotrichum acutatum, Colletotrichum coccodes, Colletotrichum dematium, and Colletotrichum gloeosporioides after treatment with the VOC mixture produced by B. velezensis CE 100 [74]. VOCs of B. velezensis JRX-YG39 have caused hyphae abnormalities such as coiling and discoloration in Colletotrichum gloeosporioides (walnut and jujube anthracnose), while the pure compounds 5-nonylamine and 3-methylbutanoic acid have caused similar morphological changes followed by cell wall lysis and fracturing [75].

Hyphal swelling, cytoplasm and protoplasm aggregation, and distortion of large amounts of balloon-shaped cells were reported for *Fusarium verticillioides, Fusarium graminearum,* and *Rhizoctonia solani* after treatment with *B. mojavensis* I4, with mycelial growth inhibition in the range of 16–76% [76]. Hyphae of *Phyllosticta citricarpa* were fattened, twisted, and deformed, with a reduced amount of conidia at the lesion site on the surface of orange plants after treatment with the VOCs produced by *Bacillus* sp. ACB-65 and *Bacillus* sp. ACB-73 [31].

In *Monilinia laxa* and *Monilinia fructicola*, the hyphal membrane and cell walls were thinner and degraded, while the cytoplasmic content was completely coagulated and no organelles could be identified [62]. One of the main mechanisms of action related to the degradation of the structure of fungal cell walls and cell membrane relies on the modification of fatty acids and ergosterol content. Benzothiazole produced by *B. subtilis* CF-3 reduced the content of long-chain saturated fatty acids (trans-linoleic acid, oleic acid, and palmitic acid) in the *Monilinia fructicola* membrane, indicating decreased cell membrane unsaturation resulting in the gradual weakening of membrane fluidity and leakage of intracellular content. Furthermore, benzothiazole treatment also reduced ergosterol content in the cell wall of *Monilinia fructicola*, affecting the integrity of the fungal cell wall and thus weakening the cell membrane and material transport [77].

### 3.4. Inhibition of Different Fungal Growth Stages by Bacillus VOCs

The VOCs of *B. subtilis* G_8_ have been shown to efficiently prevent the overwintered sclerotoid germination of *Sclerotinia sclerotiorum*, which is a major mechanism for disease suppression due to reduced apothecial formation and decimated ascospore infection [47]. Liu et al. [56] have observed significant morphological changes affecting sclerotia function, including odd shape and lack of plumpness, as well as reduced mean weight of sclerotia (44.2%, 48.0%, 29.1%, and 22.4%) in treatments with VOCs produced by *Paenibacillus polymyxa* BMP-11, *B. subtilis* BL02, *B. pumilus* BSH-4, and *B. pumilus* ZB13, respectively. A higher minimal inhibitory concentration of the VOCs produced by *B. megaterium* USB2103 was required to inhibit sclerotia germination compared to mycelial growth of *Sclerotinia sclerotiorum*, except in the case of dimethyl trisulfide whose minimal inhibitory concentration has shown similar results either on mycelium growth or sclerotia germination [49]. *B. cereus* CF4-51 VOCs (2-pentadecanone, 6,10,14-trimethyl-, 1,2-benzenedicarboxylic acid bis(2-methylpropyl) ester, dibutyl phthalate, cyclododecane, and heptadecane) have shown supreme inhibition of *Sclerotinia sclerotiorum* (65.4%) in comparison with the cell free supernatant (18.2–42.4%) and lipopeptides (55.6%) by altering the expression of four genes related to sclerotia formation (*Ss-sl2*, *SOP1*, *SsAMS2*, and *SsSac1*) [66]. *B. velezensis* VM11, *B. velezensis* VM10, and *B. amyloliquefaciens* VM42 VOCs exhibited fungistatic effects toward *Sclerotinia sclerotiorum* by inhibiting sclerotia production by 62.9%, 54.7%, and 72.5%, respectively, while healthy harvested sclerotia lost their germination ability after being incubated together with 5 μL of *Bacillus* endophyte cultures [57].

*B. subtilis* PPCB001 and *B. amyloliquefaciens* PPCB004 VOCs affected the germination and germ tube elongation of *Penicillium crustosum* [42]. Inhibition of spore germination and germ tube elongation were also observed in *Penicillium digitatum* and *Penicillium italicum* in the presence of the VOCs produced by *B. amyloliquefaciens* JBC36, which resulted in 57.8% and 54.1% in vitro inhibition of mycelial growth of *Penicillium digitatum* and *Penicillium italicum*, respectively, as well as a reduced incidence of green and blue mold on wounded mandarin fruits with control efficacies of 88% and 80.2%, respectively [78]. Inhibition of conidial germination of *Thielaviopsis paradoxa* ranged from 12% to 68.8% during the 9-h exposure to the VOCs of *B. siamensis* N-1 [79].

Reduced sporification was observed in *Aspergillus flavus* in the presence of *B. cereus* SV40, *B. subtilis* SV36/2, and *B. coagulans* SV95 VOCs [1]. A significant decrease in spore density of *Fusarium verticillioides* and *Fusarium graminearum* was observed after treatment with *B. mojavensis* I4 VOCs [76]. The different concentrations of 1%, 3%, 5%, 7%, and 10% of the VOCs produced by *B. velezensis* CE 100 reduced the spore germination of *Colletotrichum gloeosporioides* (walnut and jujube antrachnose) by 15.4%, 18.9%, 25.4%, 30.0%, and 36.4%, respectively, with remarkably reduced germ tube elongation [75]. Incompact and irregular structures of spores of *Verticillium dahliae*, causing cotton *Verticilium* wilt, together with visible holes on the spores’ surface, appeared after treatment using styrene produced by *Bacillus* sp. T6 [64]. *B. velezensis* SBB VOCs (2-nonanol—0.06 μL/mL, 2-heptanone—0.3 μL/mL, 6-methyl-2-heptanone—0.3 μL/mL, and 2-nonanone—0.6 μL/mL) completely inhibited *Verticillium dahliae* growth in vitro and inhibited production of conidia and microsclerotia [80]. Phenylacetic acid and methylphenyl acetate produced by *B. mycoides* BM02 suppressed spore germination but had no effects on the hyphal growth of *Fusarium oxysporum* f. sp. *lycopersici* in tomato [81]. Deformed and collapsed spores of *Alternaria alternata*, causing tobacco brown spot, resulted from treatment with 2-methylbutanoic acid and 3-methylbutanoic acid produced by *B. siamensis* LZ88, while inhibition of spore germination was achieved with EC_50_ values of 139.63 mg/mL and 88.07 mg/mL for the aforementioned VOCs, respectively [69].

Damaged conidial internal structures with collapsed and shrunken small vesicles, extracellular secretions around the conidial cell-wall surface, and larger lipid droplets within the conidia were observed in *Alternaria solani* after treatment with 6-methyl-2-heptanone (EC_50_ value of 10.88 μL) produced by *B. subtilis* ZD01 [68]. The germination rate of the morphologically disrupted conidia was lowered to 75%, and these conidia formed irregular (shorter) germ tubes that were not able to penetrate and invade host epidermal cell junctions. This was also confirmed by the down-regulation of the expression of two genes: the *slt2* gene, involved in mycelial growth, penetration, and pathogenicity [71], and the *wetA* gene, involved in sporulation and conidial wall formation [68]. *B. safensis* STJP VOCs (phenol, 2,4-bis (1,1-dimethylethyl)-, 3-hexadecanol, pyrrolo (1,2-a)pyrazine-1,4-dione, hexahydro-3-(2-methyl-propyl)-, 5,10-diethoxy-2,3,7,8-tetrahydro-1H,6H-dipyrrolo(1,2-a:10,20-d)pyrazine and hexadecanoic acid) have completely inhibited spore formation and conidia germination of *Alternaria alternata* [82]. The VOCs produced by *B. subtilis* ZD01 can inhibit the conidia germination (19.2%) and reduce the lesion areas and number of *Alternaria solani* in vivo significantly, which was further confirmed by the down-regulation of transcriptional expression of the *slt2* gene, a key gene that regulates the mycelial growth, penetration, sporulation, and virulence in vivo in *Alternaria solani* [71]. In the study by Xie et al. [83], 2-heptanone and isopentyl acetate produced by *B. subtilis* DZSY21 strongly inhibited the sporulation and germination of *Curvularia lunata* (maize leaf spot) conidia, while 2-methylbutyric acid inhibited sporulation, but did not affect germination. Furthermore, these VOCs repressed expression of the virulence-associated genes *clk1* and *clm1*, which encod mitogen-activated protein kinases required for conidia sporulation and pathogenicity in *Curvularia lunata* [83].

### 3.5. Prevention of Fungal Plant Attachment and Colonization by Bacillus VOCs

The VOCs of *B. subtilis* C9, with a special emphasis on DG4 (an isomer of acetyl butanediol) as an antifungal compound, have significantly reduced the incidence of stem-segment colonization by *Rhizoctonia solani* in Zoysia grass [48]. Sharifi and Ryu [84] have concluded that VOCs produced by *B. subtilis* GB03 might interfere with mycelial attachment to the hydrophobic cuticular surface of the *Arabidopsis* leaves, hence causing epiphytic mycelial growth of *Botrytis cinerea* and its inability to penetrate and colonize host tissue. Castro et al. [85] have made a relation between *B. velezensis* XT1 VOCs and the reduced number of *Verticillium dahliae* microsclerotia in the soil. *B. velezensis* OEE1 VOCs supported the reduction of *Verticillium dahliae* microsclerotia density in the naturally infested soil around olive trees [86]. The VOCs produced by *B. amyloliquefaciens* UQ154, *B. velezensis* UQ156, and *Acinetobacter* sp. UQ202 (isovaleraldehyde, 2-ethylhexanol, 2-heptanone, benzyl alcohol, and 3-methylbutanol) in a concentration of 10 μg/mL inhibited sporangia production and zoospore motility of *Phytophthora capsici* [87].

### 3.6. Altering the Expression of Genes Related to Pathogenicity, Metabolism and Antioxidant Activity of Fungal Pathogens

Zhang et al. [64] have reported the down-regulation of genes related to transport and catabolism, cell growth, and biosynthesis, especially peptidases, lipases, proteases, and chitinases, which act as plant cell wall-degrading enzymes, as well as methionyl-tRNA synthetases, in *Verticillium dahliae*, causing *Verticillium* cotton wilt, while the gene expression was modulated via styrene produced by *Bacillus* sp. T6. 

Wang et al. [88] investigated differentially expressed genes and proteins in *Colletotrichum gloeosporioides* after treatment with *B. subtilis* CF-3 VOCs. The results revealed significant down-regulation of expression of genes related to cell membrane fluidity, cell wall integrity, energy metabolism, and production of cell wall-degrading enzymes, with a special emphasis on the biosynthesis of unsaturated fatty acids and ergosterol as the significant components of cell membranes, where 2,4-di-tert-butylphenol has been detected as the major antifungal VOC [88]. *B. subtilis* CF-3 VOC 2,4-di-tert-butylphenol can inhibit the activity of the pathogenic enzymes (pectinase and cellulase) secreted by *Colletotrichum gloeosporioides* to reduce the decomposition of plant tissues in litchi fruits [89], as well as benzothiazole with the same mechanism of antifungal activity against *Monilinia fructicola* in peaches [77]. The VOCs of *Bacillus* endophytes (*B. velezensis* VM11, *B. velezensis* VM10, and *B. amyloliquefaciens* VM42) induced strong ROS (reactive oxygen species) production in *Sclerotinia sclerotiorum* mycelial cells [57]. Down-regulation of the *SOD* gene, which plays a significant role in the SOD (superoxide dismutase) synthetic pathway in *Alternaria solani*, was found as a consequence of in vivo treatment by the VOCs produced by *B. subtilis* ZD01, suggesting the inhibition of the pathogen’s antioxidant metabolism [71]. ROS can damage DNA replication and cell membranes, leading to cell death. In the study by Xie et al. [83], isopentyl acetate produced by *B. subtilis* DZSY21 caused ROS accumulation in the conidia of *Curvularia lunata* (maize leaf spot), while 2-methylbutyric acid and 2-heptanone did not affect ROS accumulation in conidia and mycelia. Here, it is suggested that these VOCs target different germination-related processes in *Curvularia lunata* conidia [83].

### 3.7. ISR Induced by Bacillus VOCs as Antifungal Mechanism of Action

One of the ways to induce resistance in plants against pathogens is physical inhibition of pathogen entrance to plant inner tissues by closing stomata. Acetoin and 2,3-butanediol as VOCs of *B. amyloliquefaciens* FZB42 modulated stomatal closure in *Arabidopsis thaliana* and *Nicotiana benthamiana*, where root absorption of VOCs was more effective than volatilization considering that VOC concentration of 250 µL was enough for stomata closure via root treatment, while 1 mM of VOCs was required for volatilization treatment. Furthermore, these VOCs have been successful in targeting pathogen entry points into hosts by triggering salicylic acid and abscisic acid signaling pathways and inducing the accumulation of hydrogen peroxide and nitric oxide, which are required for stomata closure [90]. Sharifi and Ryu [84] have found that the optimized concentration of *B. subtilis* GB03 that did not directly inhibit fungal growth of *Botrytis cinerea* successfully protected *Arabidopsis* from fungal infection, which indicates that ISR elicited by the bacterial VOCs has a more important role in biocontrol than direct inhibition of fungal growth on *Arabidopsis* plants. It was further confirmed that the ISR proportion was 90.63% and direct inhibition of fungi was 9.36% of the overall biocontrol activity, which means that ISR had the main role in suppressing *Botrytis cinerea* on *Arabidopsis* plants in conditions of low VOCs concentration. An almost three-fold increase in expression levels of plant defensin PDF1.2 indicated that the jasmonic acid signaling pathway had a key role in VOC-elicited plant defense responses, followed by the salicylic acid signaling pathway, as determined by the 2.8-fold increase in the expression level of PR1 (pathogenesis-related protein 1). On the other hand, the ethylene signaling pathway was not included in the *Arabidopsis* defense response since the expression level of the *ChiB* (basic endochitinase) gene did not display statistically significant differences between the VOCs-treated and control plants. This was also the first study to make a distinction between direct and indirect mechanisms of fungal pathogen suppression [84]. Pepper priming using the *B. velezensis* strains 5YN8 and DSN012 as VOC producers has resulted in more rapid transcription of the three pathogenicity-related genes (*NPR1*, *PR1*, and peroxidase gene), thus enhancing pepper resistance to *Botrytis cinerea* by activating the salicylic acid-mediated defense signaling pathway. In this way, gray mold biocontrol efficacy was above 50% in greenhouse experiments, with increased leaf number, stem diameter, and chlorophyll content in pepper seedlings [91]. Zheng et al. [38] have concluded that α-farnesene produced by several *Bacillus* isolates is probably related to ISR considering that it had not shown any antifungal activity in vitro against *Peronophythora litchi* while in vivo it had suppressed litchi downy blight with an efficacy of 52.34%. 

*B. siamensis* LZ88 VOCs induced plant basal immunity through the induction of defense-related enzymes against *Alternaria alternata*, including peroxidase and polyphenol oxidase, thus contributing to the reduction of brown spots in tobacco leaves [92]. *B. velezensis* XT1 VOCs increased polyphenol oxidase activity by 395%, indicating induced resistance against *Verticillium* wilt of olive (*Verticillium dahliae*) in plant tissues, resulting in reduced disease severity in young olives by almost 80% [85]. Activation of antioxidant enzymes (peroxidase, polyphenol oxidase, catalase, and superoxide dismutase) in litchi fruit to eliminate excessive reactive oxygen species to reduce plant cell damage and activate disease resistance enzymes (phenylalanine ammonia-lyase, chitinases, β-1,3-glucanase) and enhance the resistance of litchi fruits to *Colletotrichum gloeosporioides* by inhibiting its growth was observed to be enhanced by 2,4-di-tert-butylphenol produced by *B. subtilis* CF-3 [89]. Similar mechanisms of action were observed for benzothiazole produced by the same *Bacillus* isolate in the suppression of *Monilinia fructicola* peach rot [77].

### 3.8. Inhibition of Fungal Pigments Production by Bacillus VOCs

Another interesting mechanism of action of *Bacillus*-produced VOCs is the inhibition of the production of different fungal pigments. Inhibition of pigment formation by the volatiles produced by *B. subtilis* G_8_ [47], *Paenibacillus polymyxa* BMP-11, *B. subtilis* BL02, *B. pumilus* BSH-4, and *B. pumilus* ZB13 [56] was observed in *Ascochyta citrullina, Alternaria solani,* and *Alternaria brassicae*. The VOCs *of B. amyloliquefaciens* M49 inhibited the production of pink pigment by *Fusarium oxysporum* f. sp. *lactucae* [1]. *B. velezensis* SBB VOCs (2-nonanol, 2-heptanone, 6-methyl-2-heptanone, and 2-nonanone) inhibited melanin production by *Verticillium dahliae* [80]. Melanin plays an important role in providing the mechanical strength required for germ tubes, obtained by conidia germination, to penetrate host tissues [93]. The VOCs produced by *B. subtilis* DZSY21 caused inhibition of the expression of *SCD* and *brn1* genes involved in the synthesis of melanin in *Curvularia lunata* (maize leaf spot), where the inhibitory effect of isopentyl acetate was higher than that of 2-heptanone [83].

### 3.9. In Vivo Application of Bacillus VOCs in Biocontrol of Fungal Diseases

#### 3.9.1. Fungal Diseases Caused by *Colletotrichum gloeosporioides*

Zheng et al. [94] have reported successful inhibition of *Colletotrichum gloeosporioides*, the causal agent of mango anthracnose, in vitro (88.87% and 80.07%) and in vivo (94.28% and 87.06%) using the VOCs produced by *B. pumilus* TB09 *and B. thuringiensis* TB72, respectively. The main identified bioactive VOCs and their minimal inhibitory concentrations for mycelial growth inhibition were 2-nonanone, b-benzeneethanamine, 2-decanone, and 2-methylpyrazine in a concentration of 100 µL/L and thymol in a concentration of 50 µL/L. The disease incidence of mango fruit anthracnose, caused by *Colletotrichum gloeosporioides* and treated by *B. siamensis* N-1, was reduced by 44.6%, while the litchi fruit disease index and browning index were reduced by 57.8% and 82.3% through mediation by the VOCs [73].

#### 3.9.2. Fungal Diseases Caused by *Fusarium* spp.

Different *Bacillus* strains produced VOCs that inhibited *Fusarium solani*, including *B. amyloliquefaciens* subsp. *plantarum* YAU B9601-Y2, *Bacillus* spp. 041, 285, 033, 355 and *B. subtilis* XF-1, which had shown exceptionally strong inhibitory activities (75–82% inhibition), as well as *B. velezensis* FZB42 and *B. subtilis* 168, whose VOCs demonstrated 67% and 56% inhibition, respectively [95]. *B. subtilis* IBFCBF-4 VOCs successfully inhibited the mycelial growth of *Fusarium oxysporum* f. sp. *niveum* in vitro by 47.9%, while the reduction of *Fusarium* wilt in watermelon in greenhouse experiments was 51.1% [59]. *B. amyloliquefaciens* L3 VOCs (2-nonanone and 2-heptanone) were found to suppress *Fusarium* wilt of watermelon (*Fusarium oxysporum* f. sp. *niveum*) in greenhouse pot experiments, while acetoin and 2,3-butanediol were responsible for watermelon plant growth promotion [96]. Greenhouse experiments confirmed the efficacy of *B. mojavensis* I4 VOCs in the suppression of *Fusarium verticillioides, Fusarium graminearum,* and *Rhizoctonia solani* in *Arabidopsis thaliana* plants, with significant improvements in plant growth, biomass production, and chlorophyll content [76]. The VOCs of *B. cereus* MH778713 (hentriacontane and 2,4-di-tert-butylphenol) reduced the disease severity of tomato *Fusarium* wilt (*Fusarium oxysporum*) from 88.1 ± 4.1% to only 23 ± 8.2%, followed by a several-fold increase in tomato root and shoot length as well as in the fresh and dry weight of plants in treatment with 50 μg of the bioactive VOCs [97]. *In-planta* assays showed that *B. mycoides* BM02 treatment mediated by phenylacetic acid and methylphenyl acetate reduced spore attachment and germination of *Fusarium oxysporum* f. sp. *lycopersici* and increased the formation of swollen hyphae, thus protecting tomato seedlings against *Fusarium* wilt [81].

#### 3.9.3. Fungal Diseases Caused by *Sclerotinia* spp.

Shifa et al. [98] were the first to report the production of tridecane by *B. subtilis* G-1, which participated in the suppression of *Sclerotium rolfsii*, which causes stem rot or white mold of groundnut. Wu et al. [99] have identified toluene, phenol, and benzothiazole as the main VOCs produced by *B. amyloliquefaciens* NJZJSB3, which have shown comparable effects to chemical fungicides in terms of reduction of canola stem rot incidence (by 83.3%), caused by *Sclerotinia sclerotiorum,* in pot experiments. Southern blight of *Aconitum carmichaelii* Debx., caused by *Sclerotium rolfsii*, was successfully inhibited (30% with a long-acting duration of up to 62 days) in a field trial by fermentation culture VOCs of *B. subtilis* JY-7-2L [100].

#### 3.9.4. Fungal Diseases Caused by *Monilinia* spp.

Gotor-Vila et al. [30] have identified 1,3 pentadiene, acetoin (3-hydroxy-2-butanone), and thiophene as the main VOCs produced by *B. amyloliquefaciens* CPA-8, whereas thiophene was the most effective VOC in the suppression of *Monilinia laxa*, *Monilinia fructicola,* and *Botrytis cinerea* in vitro. On the other hand, during in vivo tests, it has not shown a biocontrol effect as a pure compound, while the mixture of VOCs decreased the disease incidence of cherry decay to 25% (compared to 65% in the control). The efficacy of VOCs produced by *B. amyloliquefaciens* SF14, *B. amyloliquefaciens* SP10, *Alcaligenes faecalis* ACBC1, and *Pantoea agglomerans* ACBP1 against *Monilinia fructigena* and *Monilinia laxa* causing apple brown spot was confirmed in a semi-commercial large-scale trial, with efficacy comparable to the commercial biocontrol agents (*B. subtilis* Y1336 and *Pantoea agglomerans* P10c) [101]. *B. subtilis* CF-3 VOCs combined with heat treatment could significantly reduce the rot index of peach and litchi fruit caused by *Monilinia fructicola* and *Colletotrichum gloeosporioides*, respectively, and effectively maintained fruit firmness and soluble solids content, reducing the fruit weight loss [102].

#### 3.9.5. Fungal Diseases Caused by *Alternaria* spp.

*B. velezensis* ZJ1 VOCs 2-nonanol and isooctanol showed well in vitro antifungal activity and in vivo biocontrol effect against the two pathogens causing tomato early blight and gray mold, with 40.58% and 50.90% for *Alternaria solani* and *Botrytis cinerea*, respectively, while the highest decrease in disease incidence (74.16%) was observed in 2-nonanol treatment (25 μL/mL) [70]. The VOCs produced by *B. velezensis* L1 considerably decreased the wolfberry’s disease index and decay incidence *in vivo*, caused by *Alternaria iridiaustralis*, where no mycelial growth was observed on the wolfberry fruits after exposure to the 8 LB (Luria-Bertrani) plates. The leading VOC with the strongest antifungal effect was 2,3-butanedione, which totally inhibited *Alternaria iridiaustralis* in wolfberry fruit at a concentration of 60 μL/L [73]. The VOCs produced by *B. subtilis* ZD01 significantly reduced lesion diameter in potato leaves, as well as the population density of *Alternaria solani* (early blight pathogen), with acetophenone, 2-nonanone, *m*-tolunitrile, 2-ethylhexanol, 2-heptanone, benzylacetone, 6-methyl-2-heptanone, benzothiazole, 5-methyl-2-hexanone, aniline, 4-methylanisole, benzoxazole, valerophenone, and 2,5-dimethylpyrazine as the major antifungal VOCs [68].

#### 3.9.6. Fungal Diseases Caused by *Botrytis cinerea*

Dibutyl phthalate produced by *B. velezensis* JRX-YG39 has significantly reduced disease incidence in *Arabidopsis thaliana* caused by *Botrytis cinerea* up to 19.38% [103]. Zhang et al. [41] have reported 2,6-di-tert-butyl-4-methylphenol (BHT) and 2,4-di-tert-butylphenol (2,4-DTBP) produced by *B. siamensis* G-3 as biofumigants for controlling raspberry postharvest diseases caused by *Botrytis cinerea* and *Rhizopus stolonifer* in vivo, with biocontrol efficacy of 52.38% and 93.33%, respectively. This results in the possibility of storing raspberries for 20 days at 0 °C with the disease rate maintained at ~10% after the 2,4-DTBP treatment. Diacetyl and benzaldehyde produced by *B. velezensis* strains BUZ-14, I3, and I5 have been reported as promising VOCs for active packaging during the postharvest commercialization of fruit. Furthermore, VOCs of *B. velezensis* I3 suppressed gray mold (*Botrytis cinerea*) in grapes by 50%, while the VOCs of *B. velezensis* BUZ-14 decreased brown rot severity in apricots (*Monilinia fructicola*) from 60 to 4 mm. Diacetyl was shown as suitable for biocontrol of gray mold with a concentration of only 0.02 mL/L and blue rot in mandarins at the same dose up to 60% [35].

#### 3.9.7. Suppression of Mycotoxigenic Fungi by *Bacillus*-Based VOCs

*B. megaterium* BM344-1 VOCs (hexadecanoic acid methyl ester (palmitic acid) and tetracosane) have caused 51% inhibition of *Aspergillus flavus* growth in in vivo experiments on maize kernels, as well as a reduction in aflatoxin synthesis from 91.81 ± 29.10 μg/kg to 25.34 ± 6.72 μg/kg. On the other hand, in vitro experiments have resulted in significant growth inhibition of mycotoxigenic fungi *Penicillium verrucosum* (66.7%), *Aspergillus flavus* (29.4%), and *Fusarium verticillioides* (18.2%), as well as in complete inhibition of aflatoxins (AFB1, AFG1, and AFG2), ochratoxin A, and fumonisin B1 (FB1) synthesis on artificial media [104]. In vivo assays on maize ears resulted in an 88% reduction in *Aspergillus flavus* growth and complete inhibition of fungal sporulation and aflatoxin accumulation by the VOCs produced by *B. licheniformis* BL350-2, dominated by 3-methyl-1-butanol [105].

#### 3.9.8. Suppression of Other Fungal Diseases by *Bacillus*-Based VOCs

VOCs emitted by bacterial antagonists *B. amyloliquefaciens* UQ154, *B. velezensis* UQ156, and *Acinetobacter* sp. UQ202 negatively influenced the mycelial growth of the soil-borne phytopathogenic oomycete *Phytophthora capsici* by 35%, with significant positive effects on the increase in chili biomass (shoot and root fresh weights) and the primary root length, as well as the promotion of both root hair growth and lateral root development. The most important VOCs with antifungal activity were isovaleraldehyde, 2-ethylhexanol, 2-heptanone, benzyl alcohol, and 3-methylbutanol [87]. The leaf spot disease indexes of maize leaves sprayed with conidia of *Curvularia lunata* treated with the VOCs produced by *B. subtilis* DZSY21 were reduced from 60.52 to 26.64% [83]. In vivo application of 2,3-butanedione and 3-methylbutyric acid produced by *B. subtilis* CL2 significantly reduced the weight loss rate of wolfberry fruits caused by the pathogenic fungus *Mucor circinelloides*, as well as the decay incidence rate caused by *Fusarium arcuatisporum, Alternaria iridiaustralis*, and *Colletotrichum fioriniae* [72]. The VOCs produced by *B. methylotrophicus* BCN2 and *B. thuringiensis* BCN10 played complementary roles in controlling *Fusarium oxysporum*, *Botryosphaeria* sp., *Trichoderma atroviride, Colletotrichum gloeosporioides,* and *Penicillium expansum*, providing freshness to loquat fruits for ten days with a disease incidence of 20.19% compared to 54.17% in the control group [106]. The VOCs produced *Bacillus* sp. ACB-65 and *Bacillus* sp. ACB-73 cultured in TSB culture medium provided 86% inhibition of freckles that evolved into hard spots, with more superficial citrus black spot (*Phyllosticta citricarpa*) lesions in oranges [31]. The importance of the applied VOC concentration was pointed out by Zheng et al. [38], who discovered that 1-(2-aminophenyl)ethanone, benzothiazole, and α-farnesene had shown the highest efficacy in suppression of litchi downy blight (*Peronophythora litchi*) when applied in the lowest concentration (100 mg/L) from the examined concentration range (100–1000 mg/L). It suggested that VOCs in small concentrations could achieve the priming effect in plants, thus sensitizing plants for faster and/or stronger responses to successive pathogen attacks by acting as signaling molecules for environmental stresses. 

**Table 2:** Literature overview concerning antifungal VOCs produced by *Bacillus* spp.

*Bacillus* Strain	Plant Pathogen (Disease)	Antifungal VOCs	Reference
*B. subtilis* 155	*Penicillium digitatum* Sacc. (green mold of citrus fruit)	mixture of VOCs	Leelasuphakul et al. [43]
*B. subtilis* G_8_	*Sclerotinia sclerotiorum* *Botrytis cinerea* *Alternaria brassicae* *Alternaria solani* *Alternaria citrulina* *Fusarium oxysporum* *Cercospora kikuchii Chupp* *Rhizoctonia solani*	mixture of VOCs	Liu et al. [47]
*B. subtilis*	*Sclerotinia sclerotiorum* (white mold of *Lactuca sativa*)	mixture of VOCs	Monteiro et al. [58]
*B. subtilis* GB03	*Botrytis cinerea*	mixture of VOCs	Sharifi and Ryu [84]
*B. subtilis* ACB-AP3 *B. subtilis* ACB-83	*Phyllosticta citricarpa* (orange black spot)	mixture of VOCs	Kupper et al. [107]
*B. subtilis* IBFCBF-4	*Fusarium oxysporum* f. sp. *niveum* (watermellon Fusarium wilt)	mixture of VOCs	Zhu et al. [59]
*B. subtilis* JY-7-2L	*Sclerotium rolfsii* (southern blight of *Aconitum carmichaelii* Debx.)	mixture of VOCs	Zou et al. [100]
*B. subtilis* C9	*Rhizoctonia solani*	acetylbutanediol	Islam et al. [48]
*B. subtilis* G-1	*Sclerotium rolfsii* (stem rot or white mould of groundnut)	tridecane	Shifa et al. [98]
*B. subtilis* M29	*Botrytis cinerea*	1-butanol acetic acid butyl ester 1-heptylene-4-alcohol 3-methyl-3-hexanol furan-tetrahydro-2,5-dimethyl 2,6-diisocyanato-1-methyl-benzene 1-propoxy-2-propanol benzophenone	Mu et al. [61]
*B. subtilis* CF-3	*Monilinia fructicola* *Colletotrichum gloeosporioides*	1-octanol 2,4-di-tert-butylthiophenol	Gao et al., 2017 [37]
	*Botrytis cinerea* (strawberry gray mold) *Colletotrichum gloeosporioides* (litchi antrachnose) *Penicillium expansum* (blue mold of apple) *Monilinia fructicola* (peach brown rot) *Alternaria alternata* (Alternaria rot and black spot of jujube)	2,4-di-tert-butylthiophenol benzothiazole 1-octanol benzoic acid benzaldehyde 3-methylbutanal	Gao et al., 2018 [36]
	*Monilinia fructicola* *Colletotrichum gloeosporioides*	mixture of VOCs	Wu et al. [102]
	*Monilinia fructicola*	benzothiazole	Zhou et al. [77]
	*Colletotrichum gloeosporioides*	2,4-di-tert-butylphenol	Zhao et al. [89] Wang et al. [88]
*B. subtilis* CL2	*Mucor circinelloides* *Fusarium arcuatisporum* *Alternaria iridiaustralis* *Colletotrichum fioriniae*	2,3-butanedione 3-methylbutyric acid	Ling et al. [72]
*B. subtilis* BTK1	*Sarocladium oryzae* (rice sheath rot)	3-heptanone, 5-ethyl-4-methyl- butanoic acid, 2-methyl 1-propanol 2,2-dimethyl acetate	Surya et al. [108]
*B. subtilis* DZSY21	*Curvularia lunata* (maize leaf spot)	2-methylbutyric acid 2-heptanone isopentyl acetate	Xie et al. [83]
*B. subtilis* ZD01	*Alternaria solani* (potato early blight)	acetophenone 2-nonanone m-tolunitrile 2-ethylhexanol 2-heptanone benzylacetone 6-methyl-2-heptanone benzothiazole 5-methyl-2-hexanone	Zhang et al. [71]
		6-methyl-2-heptanone	Zhang et al., [68]
*B. subtilis* BS-01	*Alternaria solani* (tomato Alternaria blight)	triphenylphosphine oxide pyrrolo[1,2-a] pyrazine-1,4-dione, hexahydro-3-(2-methylpropyl) pyrrolo[1,2-a] pyrazine-1,4-dione, hexahydro-3-(phenylmethyl) n-hexadecanoic acid n-tridecan-1-ol octadecane octadecanoic acid eicosane dodecyl acrylate	Awan et al. [109]
*B. amyloliquefaciens* JBC36	*Penicillium digitatum* (green mold of citrus fruit) *Penicillium italicum* (blue mold of citrus fruit)	mixture of VOCs	Yu et al. [78]
*B. amyloliquefaciens* NJN-6	*Fusarium oxysporum* f. sp. *cubense*	phenol 2,3,6-trimethyl-phenol 2-undecanone 2-dodecanone 2-tridecanone	Yuan et al., 2012 [44]
		mixture of VOCs	Yuan et al. [110]
*B. amyloliquefaciens* W19	*Fusarium oxysporum* f. sp. *cubense* (bannana *Fusarium* wilt)	o-xylene 2-heptanone benzene, 2-propenyl benzene,1,4-dichloro undecane, 1,2-methyl acetophenone 2-nonanone nonanane 1-(4-methylphenyl)ethanone 2- decanone naphthalene 2-undecanone tridecane 2- dodecanone tetradecane 2-tridecanone pentadecane hexadecane tetradecane	Wang et al. [111]
*B. amyloliquefaciens* NJZJSB3	*Sclerotinia sclerotiorum* (canola stem rot)	toluene phenol benzothiazole	Wu et al. [99]
*B. amyloliquefaciens* CPA-8	*Monilinia laxa* *Monilinia fructicola* *Botrytis cinera* (sweet cherry decay)	1,3-pentadiene acetoin thiophene	Gotor-Vila et al. [30]
*B. amyloliquefaciens* DA12	*Fusarium asiaticum* *Fusarium graminearum* *Fusarium proliferatum* *Fusarium verticillioides* *Fusarium oxysporum* f. sp. *lycopersici* (tomato wilt) *Botrytis cinerea* (cucumber grey mould) *Colletotrichum coccodes* (pepper anthracnose) *Endothia parasitica* (chestnut blight) *Raffaelea quercus-mongolicae* (oak wilt) *Rhizoctonia solani* (rice sheath blight)	2-heptanone 5-methyl heptanone 6-methyl heptanone	Lee et al. [51]
*B. amyloliquefaciens* L3	*Fusarium oxysporum* f. sp. *niveum* (watermellon Fusarium wilt)	2-nonanone 2-heptanone	Wu et al. [96]
*B. amylolicefaciens* ALB629 *B. amylolicefaciens* UFLA285	*Colletotrichum lindemuthianum* (common bean antrachnose)	3-methylbutanoic 2-methylbutanoic acid	Martins et al. [41]
*B. amyloliquefaciens* BsA3MX *B. amyloliquefaciens* BsC11MX	*Macrophomina phaseolina* (cowpea charcoal rot)	mixture of VOCs	Rangel- Montoya et al. [112]
*B. amyloliquefaciens* D747 (Amylo-X^®^) *B. amyloliquefaciens* FZB24 (Taegro^®^) *B. amyloliquefaciens* MBI600 (Serifel^®^) *B. amyloliquefaciens* QST713 (Serenade^®^Aso)	*Plenodomus tracheiphilus* (Mal Secco disease of *Citrus volkameriana*)	mixture of VOCs	Aiello et al. [113]
*B. velezensis* 5YN8 *B. velezensis* DSN012	*Botrytis cinerea* (pepper gray mold)	mixture of VOCs	Jiang et al. [91]
*B. velezensis* NKG-2	*Fusarium oxysporum* *Fusarium graminearum* *Botrytis cinerea* *Alternaria alternata* *Fulvia fulva* *Ustilaginoidea virens*	mixture of VOCs	Myo et al. [55]
*B. velezensis* C2	*Verticillium dahliae* (tomato wilt disease)	mixture of VOCs	Dhouib et al. [63]
*B. velezensis* OEE1	*Fusarium solani*	mixture of VOCs	Cheffi et al. [114]
	*Verticillium dahliae*	cyclo (Leu-Pro)	Cheffi Azabou et al. [86]
*B. velezensis* XT1	*Verticillium dahliae* (Verticillium wilt of olive tree)	mixture of VOCs	Castro et al. [85]
*B. velezensis* RDA1	*Rosellinia necatrix*	mixture of VOCs	Sawant et al. [46]
*B. velezensis* JCK-1618 *B. velezensis* JCK-1696	*Epicoccum tobaicum* *Mycosphaerella cerasella*	mixture of VOCs	Han et al. [25]
*B. velezensis* ZSY-1	*Alternaria solani* *Botrytis cinerea* *Valsa mali* *Monilinia fructicola* *Fusarium oxysporum* f. sp. *capsicum* *Colletotrichum lindemuthianum*	pyrazine (2,5-dimethyl) benzothiazole 4-chloro-3-methyl phenol-2,4-bis (1,1-dimethylethyl)	Gao et al. [50]
*B. velezensis* G341	*Alternaria panax* (ginseng blight) *Botrytis cinerea* (tomato gray mold) *Colletotrichum coccodes* (red pepper antrachnose) *Fusarium oxysporum* f. sp. *lycopersici* (tomato *Fusarium* wilt) *Magnaporthe oryzae* (rice blast) *Phytophthora capsici* *Pythium infestans* (tomato late blight) *Pythium ultimum* (cucumber damping-off) *Rhizoctonia solani* (rice sheath blight) *Sclerotinia sclerotiorum* (cucumber Sclerotinia rot)	dimethylsulfoxide 1-butanol acetoin	Lim et al. [54]
*B. velezensis* BUZ-14 *B. velezensis* I3 *B. velezensis* I5	*Monilinia fructicola* *Monilinia laxa* *Penicillium italicum* *Botrytis cinerea*	benzaldehyde diacetyl	Calvo et al. [115]
*B. velezensis* CT32	*Verticillium dahliae* *Glomerella cingulata* *Thanatephorus cucumeris* *F. oxysporum* f. sp. *cucumerinum* *F. oxysporum* f. sp. *fragariae* *F. oxysporum* f. sp. *niveum* *Botryosphaeria dothidea* *Botrytis cinerea*	decanal benzothiazole 3-undecanone 2-undecanone 2-undecanol undecanal 2,4-dimethyl-6-tert-butylphenol	Li et al. [116]
*B. velezensis* CE 100	*Colletotrichum gloeosporioides* (walnut and jujube antrachnose)	5-nonylamine 3-methylbutanoic acid	Choub et al. [75]
	*Colletotrichum acutatum* *Colletotrichum coccodes* *Colletotrichum dematium* *Colletotrichum gloeosporioides*	mixture of VOCs	Kim et al. [74]
*B. velezensis* JRX-YG39	*Botrytis cinerea* *Fusarium pernambucanum* *Alternaria alternata* *Colletotrichum gloeosporioides*	dibutyl phthalate	Feng et al. [103]
*B. velezensis* L1	*Alternaria iridiaustralis* *Phytophthora capsici* *Colletotrichum capsici* *Fusarium oxysporum* *Fusarium graminearum* *Fusarium annulatum* *Fusarium arcuatisporum* *Botrytis cinerea* *Rhizoctonia solani* *Talaromyces tumuli* *Colletotrichum fioriniae*	2,3-butanedione	Ling et al. [73]
*B. velezensis* ZJ1	*Alternaria solani* (tomato early blight) *Botrytis cinerea* (tomato gray mold)	isooctanol 2-nonanol	Ren et al. [70]
*B. velezensis* HY-3479	*Colletotrichum acutatum* (pepper ripe rot) *Cylindrocarpon destructans* (ginseng root rot) *Rhizoctonia solani* (pepper damping-off) *Sclerotinia sclerotiorum* (pepper white mold)	3-methyl-1-butanol (R, R)-2,3-butanediol acetoin benzoic acid	Song et al. [117]
*B. velezensis* SBB	*Verticillium dahliae*	2-nonanol 2-heptanone 6-methyl-2-heptanone 2-nonanone	Wang et al. [80]
*B. licheniformis* BL350-2	*Aspergillus westerdijkiae* *Aspergillus carbonarius* *Aspergillus niger* *Aspergillus flavus* *Aspergillus parasiticus* *Aspergillus ochraceus* *Penicillium verrucosum*	3-methyl-1-butanol	Ul Hassan et al. [105]
*B. pumilus* TM-R	*Alternaria alternata* *Cladosporium cladosporioides* *Curvularia lunata* *Fusarium oxysporum* *Penicillium italicum*	methyl isobutyl ketone ethanol 5-methyl-2-heptanone S-()-2-methylbutylamine	Morita et al. [34]
*B. megaterium* BmBP17	*Phytophthora capsici* *Magnaporthe oryzae*	pyrazine, 2-ethyl-3-methyl pyrazine, 2-ethyl- pyrazine, 2,5-dimethyl pyrazine, 2-methyl	Munjal et al. [27]
*B. megaterium* BM344-1	*Aspergillus flavus* *Aspergillus carbonarius* *Penicillium verrucosum* *Fusarium verticillioides*	hexadecanoic acid methyl ester (palmitic acid) tetracosane	Saleh et al. [104]
*B. mycoides*	*Rhizoctonia solani* Kühn *Pythium aphanidermatum* Edson (cabbage damping-off)	dimethyl disulphide ammonia	Huang et al. [29]
*B. mycoides* BM02	*Fusarium oxysporum* f. sp. *lycopersici* (tomato *Fusarium* wilt)	phenylacetic acid methylphenyl acetate	Wu et al. [81]
*B. atrophaeus* CAB-1	*Botrytis cinerea* (tomato gray mold) *Sphaerotheca fuliginea* (cucumber powdery mildew)	O-anisaldehyde	Zhang et al. [60]
*B. atrophaeus* JZB120050	Botrytis cinerea *Fusarium oxysporum* f. sp. *conglutinans* *Fusarium oxysporum* f. sp. *niveum* *Fusarium oxysporum* f. sp. *vasinfectum* *Fusarium solani* f. sp. *pisi* *Fusarium oxysporum Schlecht* *Fusarium graminearum* *Rhizoctonia cereal* *Gaeumannomyces graminis* *Monilinia fructicola* *Botryosphaeria dothidea* *Colletotrichum gloeosporioides*	mixture of VOCs	Ni et al. [28]
*B. atrophaeus* HAB-5	*Colletotrichum gloeosporioides*	chloroacetic acid tetradecyl esters octadecane hexadecanoic acid, methyl ester	Rajaofera et al. [32]
*B. cereus* MH778713	*Fusarium oxysporum* (tomato *Fusarium* wilt)	hentriacontane 2,4-di-tert-butylphenol	Ramírez et al. [97]
*B. cereus* CF4-51	*Sclerotinia sclerotiorum*	2-pentadecanone, 6,10,14-trimethyl- 1,2-benzenedicarboxylic acid bis(2-methylpropyl) ester dibutyl phthalate cyclododecane heptadecane	Hu et al. [66]
*B. mojavensis* I4	*Fusarium verticillioides* *Fusarium graminearum* *Rhizoctonia solani*	mixture of VOCs	Ghazala et al. [76]
*B. siamensis* G-3	*Botrytis cinerea* *Rhizopus stolonifer*	2,6-di-tert-butyl-4-methylphenol 2,4-di-tert-butylphenol	Zhang et al. [39]
*B. siamensis* N-1	*Colletotrichum gloeosporioides* *Glomerella sp.* *Pestalotiopsis microspora* *Diaporthe phaseolorum* *Phomopsis sp.* *Diaporthe phaseolorum* *Geotrichum candidum* *Fusarium lateritium* *Fusarium oxysporm* *Fusarium equiseti* *Fusarium incarnatum* *Fusarium *sp. *Lasiodiplodia theobromae* *Phomopsis caricae-papayae* *Thielaviopsis paradoxa*	1-undecene 3-methyl-1-butanol 2-nonanone 1,3,5,7-cyclooctatetraene phenol	You et al. [79]
*B. siamensis* LZ88	*Alternaria alternata* (tobacco brown spot)	mixture of VOCs	Xie et al. [92]
		2-methylbutanoic acid 3-methylbutanoic acid	Wang et al. [69]
*B. safensis* STJP	*Alternaria alternata*	phenol, 2,4-bis (1,1-dimethylethyl)-3-hexadecanol pyrrolo (1,2-a)pyrazine-1,4-dione hexahydro-3-(2-methyl-propyl)- 5,10-diethoxy-2,3,7,8-tetrahydro-1H,6H-dipyrrolo(1,2-a:10,20-d)pyrazine hexadecanoic acid	Prakash and Arora [82]
*Bacillus* sp. B44	*Fusarium oxysporum* f. sp. *lycopersici*	mixture of VOCs	Jangir et al. [40]
*Bacillus* sp. ACB-65 *Bacillus* sp. ACB-73	*Phyllosticta citricarpa* (orange black spot)	mixture of VOCs	Fujimoto et al. [31]
*Bacillus* sp. T6	*Verticillium dahliae* (cotton *Verticillium* wilt)	styrene	Zhang et al. [64]
*Bacillus* sp. LPPC170	*Fusarium kalimantanense* (Panama disease of bannana)	acetic acid propanoic acid butanoic acid valeric acid isovaleric acid	de Ávila Santos et al. [67]
*B. subtilis* XF-1 *B. amyloliquefaciens* subsp. *plantarum* FZB42 *B. amyloliquefaciens* subsp. *plantarum* YAU B9601-Y2 *B. subtilis* 168 *Bacillus* spp. strains 033, 041, 355 and 285	*Fusarium solani*	mixture of VOCs	Li et al. [97]
*B. cereus* KY094642 *B. safensis* KY094643	*Alternaria* sp. (leaf spot and blight disease of lentils)	mixture of VOCs	Roy et al. [118]
*B. amyloliquefaciens* RS-25 *B. licheniformis* MG-4 *B. subtilis* Z-14 *B. subtilis* Pnf-4	*Botrytis cinerea* (tomato, strawberry, and grapefruit gray mold)	mixture of VOCs	Chen et al. [119]
*B. methylotrophicus* BCN2 *B. thuringiensis* BCN10	*Fusarium oxysporum* *Botryosphaeria sp.* *Trichoderma atroviride* *Colletotrichum gloeosporioides* *Penicillium expansum*	mixture of VOCs	He et al. [106]
*B. velezensis* BUZ-14 *B. ginsengihumi* S38	*Botrytis cinerea*	mixture of VOCs	Calvo et al. [35]
*B. mycoides* *B. subtilis* *B. thuringiensis*	*Aspergillus ochraceus* *Aspergillus westerdijkiae* *Aspergillus flavus* *Aspergillus parasiticus*	mixture of VOCs	Hlebová et al. [53]
*B. safensis* RGM 2450 *B. siamensis* RGM	*Botrytis cinerea* *Colletotrichum acutatum* *Fusarium oxysporum* *Phytophtora cinnamomi*	mixture of VOCs	Altimira et al. [120]
*B. subtilis* PPCB001 *B. amyloliquefaciens* PPCB004	*Penicillium digitatum* Sacc. *Penicillium italicum* Wehmer *Penicillium crustosum* Thom	acetoin	Arrebola et al. [42]
*B. pumilus* TB09 *B. thuringiensis* TB72	*Colletotrichum gloeosporioides* (mango antrachnose)	2-nonanone b-benzeneethanamine 2-decanone thymol 2-methylpyrazine	Zheng et al. [94]
*B. subtilis* *B. amyloliquefaciens* *B. cereus*	*Aspergillus niger* *Aspergillus flavus* *Aspergillus parasiticus* *Aspergillus clavatus* *Fusarium oxysporum* f.sp. *lactucae Moniliophthora perniciosa*	propanone 1-butanol 3-methyl-1-butanol acetic acid 2-methylpropanoic acid carbon disulphide 3-methylbutanoic acid ethyl acetate	Chaves-Lopez et al. [1]
*B. vallismortis* 12a *B. altitudinis* 14b	*Monilinia fructicola* (peach brown rot)	6-methyl-2-heptanone 2-pentylfuran cedrol isodecyl methacrylate	Liu et al. [65]
*B. velezensis* VM11 *B. velezensis* VM10 *B. amyloliquefaciens* VM42	*Sclerotinia sclerotiorum*	2-undecanone 1,3-butadiene benzothiazole N,N-dimethyldodecylamine	Massawe et al. [57]
*B. amyloliquefaciens* HA *B. stratosphericus* SO *B. acidiceler* SJJ *B. mycoides* HB	*Fusarium solani* *Fusarium *sp. *Colletotrichum gloeosporioides* *Phytophthora cinnamomi*	2,3,5-trimethylpyrazine 2-nonanone 2-decanone 2-dodecanone dimethyl disulfide dimethyl trisulfide	Guevara-Avendaño et al. [121]
*B. nakamurai* *B. pseudomycoides* *B. proteolyticus* *B. thuringiensis*	*Botrytis cinerea*	2-heptanone dodecanal dimethyl disulfide dimethyl trisulfide 3-methylbutan-1-ol	Chaouachi et al. [122]
*Paenibacillus polymyxa* BMP-11 *B. subtilis* BL02 *B. pumilus* BSH-4 *B. pumilus* ZB13	*Sclerotinia sclerotiorum* *Botrytis cinerea* *Alternaria brassicae* *Alternaria solani* *Ascochyta citrullina* *Fusarium oxysporum* *Cercospora kikuchii Chupp* *Rhizoctonia solani* *Phoma arachnidicola* *Verticillium dahiae* *Fusarium graminerum*	mixture of VOCs	Liu et al. [56]
*Pseudomonas fluorescens* Pf 9A-14 *Pseudomonas sp.* Psp. 8D-45 *B. subtilis* Bs 8B-1	*Pythium capsici* (cucumber damping-off) *Phytophthora capsici* (cucumber root rot) *Rhizoctonia solani* (radish damping-off)	mixture of VOCs	Khabbaz et al. [45]
*B. megaterium* KU143 *Pseudomonas protegens* AS15	*Aspergillus flavus*	mixture of VOCs	Mannaa et al. [52]
*Pichia kudriavzevii* *Candida labiduridarum* *B. acidiceler* *B. macauenses* *B. amyloliquefaciens* *B. pumilus*	*Sclerotinia sclerotiorum*	mixture of VOCs	Cavalcanti et al. [123]
*B. amyloliquefaciens* SF14 *B. amyloliquefaciens* SP10 *Alcaligenes faecalis* ACBC1 *Pantoea agglomerans* ACBP1	*Monilinia fructigena* *Monilinia laxa* (apple brown rot)	mixture of VOCs	Lahlali et al. [101]
*Pseudomonas brassicacearum* *Pseudomonas putida* *B. megaterium*	*Botrytis cinerea* *Phytophthora nicotianae* *Rhizoctonia solani* *Sclerotinia sclerotiorum* *Verticillium dahliae* *Fusarium oxysporum* *Macrophomina phaseolina*	acetic acid 2-nonanone dimethyl trisulfide	Giorgio et al. [49]
*Bacillus* spp. *Paenibacillus* spp.	*Rhizoctonia solani* *Fusarium graminearum* *Phytophthora capsici* *Pythium aphanidermatum* *Podosphaera fuliginea*	acetoin diacetyl	Khalaf and Raizada [124]
*B. amyloliquefaciens* LI24 *B. amyloliquefaciens* PP19 *B. licheniformis* HS10 *B. pumilus* PI26 *Exiguobacterium acetylicum* SI17	*Peronophythora litchii* (litchi downy blight)	1-(2-aminophenyl)ethanone benzothiazole α-farnesene	Zheng et al. [38]
*B. amyloliquefaciens* UQ154 *B. velezensis* UQ156 *Acinetobacter sp.* UQ202	*Phytophthora capsici*	isovaleraldehyde 2-ethylhexanol 2-heptanone benzyl alcohol 3-methylbutanol	Syed-Ab-Rahman et al. [87]
*B. atrophaeus* L193 *B. velezensis* XT1 *Psychrobacillus vulpis* Z8	*Alternaria alternata* *Botrytis cinerea* *Fusarium oxysporum* *Fusarium solani* *Monilinia fructicola* *Monilinia laxa* *Sclerotinia sclerotiorum*	acetoin acetic acid 2,3-butanediol isopentanol dimethyl disulphide isopentyl isobutanoate	Toral et al. [62]

## 4. Nematicidal Action of *Bacillus*-Based VOCs

Besides the antimicrobial activity against bacterial and fungal pathogens, representatives of the *Bacillus* genus are recognized as potential biocontrol agents effective in the suppression of plant-parasitic nematodes. The overview of published literature data focused on the nematicidal activity of *Bacillus*-based VOCs is summarized and examples of active VOCs are given in Table 3. The mode of action of *Bacillus* strains is defined through several approaches, including regulation of nematode behavior, competition for nutrients, and interference with nematode-host recognition [125]. Some of the examples reported in the scientific literature include *B. firmus* YBf-10, which shows nematicidal activity against the root-knot nematode *Meloidogyne incognita*, *B. cereus* C1-7, which inhibits root gall formation and reduces egg production of the carrot and tomato parasite *Meloidogyne hapla* [126]; and *B. subtilis*, which is active even under high temperatures, making it applicable in greenhouses as a biocontrol agent of root-knot nematode [125]. The previous studies also reported that secondary metabolites produced by *Bacillus* strains including *B. megaterium*, *B. cereus, B. thuringiensis,* and *B. pumilus* are effective against *Meloidogyne exigua, Bursaphelenchus xylophilus,* and *Ditylenchus destructor*. The production of volatile metabolites was also recognized as a potential mechanism of action for nematode suppression. *B. nematocida* B16 was one of the examples reported in the literature, where it was explained that it lures nematodes by producing seven VOCs that attract worms and afterward enter the intestine of the nematodes [126].

### 4.1. Styrene as the Nematicidal Bacillus-Based VOC

In the study by Luo et al. [126], a total of 45 members of the *Bacillus* genus were isolated from the root-knot nematode-infested tomato rhizosphere soil, and 5 of them were positive in terms of nematicidal activity with a mortality rate over 60%. The strain exhibiting the highest potential of nematicidal activity, causing a 98.1% mortality rate, was identified as *B. mycoides* R2. Styrene was defined as the primary nematicidal substance produced by the investigated strain, expressing high nematicidal activity against both free-living nematodes (*Caenorhabditis elegans*) and the root-knot nematode *Meloidogyne incognita.* In comparison with other nematicides, for example, chloronicotinyl insecticide thiacloprid for the nematode *Meloidogyne incognita* J2, with the LC50 of 36.2 mg/L in tomato crops, this value for styrene was 4.55 μg/mL [126]. The additional unique advantage for the control of nematodes with this VOC in the field lies in the fact that it fully evaporates in the soil and quickly and efficiently repels nematodes from crops [126].

### 4.2. Interference of Bacillus-Based VOCs with Nematodes’ Chemotaxis

In another study, the potential nematicidal strain, *B. subtilis* Bs-1, was isolated from tomato rhizosphere and tested for in vitro nematicidal and ovicidal activities against *Meloidogyne incognita*, but the study also included efficiency evaluation in the pot experiments and in the field [127]. The results indicated high nematicidal activity, with an egg mortality rate of 100%, and a positive outcome even in field conditions. *B. subtilis* Bs-1 successfully reduced the disease index and stimulated the growth and yield of cucumber. Previous studies also confirmed that the *B. subtilis* isolate repelled second-stage juveniles (J2s) and reduced nematode production in tomatoes [127]. The major VOC produced by *B. subtilis* Bs-1 was CO_2_, and other identified active substances were acetic acid, 2-heptanone, pyrazine 2,5-dimethyl- and dimethyl disulfide, which were reported in previous studies as leading to chemotaxis in *Meloidogyne incognita* or *Caenorhabditis elegans* [128,129]. Considering the above-mentioned components’ roles, it should be findings pointed out that *Meloidogyne incognita* was attracted by low concentrations of CO_2_ and that acetic acid (0.1%) caused 100% mortality of *Meloidogyne incognita.* Organic sulfide also showed strong antimicrobial and insecticidal activities, and phenol, cyclohexanol, 2-octanol, and 2-undecanone expressed suppressive activity against nematodes [125]. The VOCs of another strain, *B. cereus* Bc-cm103, caused the mortality rates of *Meloidogyne incognita* J2s to be 90.8% and 97.2% after 24 h and 48 h of incubation, respectively. The identification of VOCs revealed the presence of 21 compounds, including alkanes, alkenes, esters, and sulfides, but the nematocidal activity was observed in the case of dimethyl disulfide (30.63%) and S-methyl ester butanethioic acid (30.29%) [130]. As it was suggested in previous studies, the mechanism of action included strong interference of VOCs with the chemotaxis of *Meloidogyne incognita* to cucumber roots.

### 4.3. Interference of Bacillus-Based VOCs with Nematodes’ Antioxidant Metabolism

Ayaz et al. [131] conducted the experiments with *Bacillus* GBSC56 isolated from the Tibet region of China investigating the potential nematicidal activity against *Meloidogyne incognita*. The VOCs profile analysis revealed the presence of 10 compounds, while 3 of them, including dimethyl disulfide, methyl isovalerate, and 2-undecanone indicated strong nematicidal activity with mortality rates of 87%, 83%, and 80%, respectively. The activity of VOCs was based on severe oxidative stress caused by nematodes, resulting in rapid death. Additionally, the activity of antioxidant enzymes SOD, CAT, POD, and APX has enhanced in *Meloidogyne incognita*-infested roots in the presence of volatiles, which might reduce the adverse effect of oxidative stress induced after infection.

### 4.4. Specific Bacillus-Based VOCs Exhibiting Nematicidal Action

*B. licheniformis* JF-22 was another *Bacillus* strain recognized as a potential biocontrol agent active against *Meloidogyne incognita*. It was isolated from the tomato rhizosphere in the area where healthy plants were grown in the presence of the tomato root-knot nematode, and the study pointed out that acetoin, 2,3-butanediol, and hexamethylcyclotrisiloxane are the main components among the produced VOCs [132]. The VOCs produced by *B. altitudinis* AMCC 1040 were analyzed in the study by Ye et al. [133] and grouped into four major categories: ethers, alcohols, ketones, and organic acids. Out of eight compounds, six exhibited different levels of nematicidal activity, including 2,3-butanedione, acetic acid, 2-isopropoxy ethylamine, 3-methylbutyric acid, 2-methylbutyric acid, and octanoic acid. All four organic acids showed strong nematicidal activity, with octanoic acid showing the highest, followed by the activity of acetic acid as the second best, while two isomeric organic acids, 2-methyl-butanoic acid and 3-methyl-butanoic acid, had the same suppressive effect. Among the two ketones tested, only 2,3-butanedione showed activity [133].

**Table 3:** Literature overview—nematicidal VOCs produced by *Bacillus* spp.

*Bacillus* Strain	Plant Pathogen	Nematicidal VOCs	Reference
*B. mycoides* R2	*Caenorhabditis elegans* *Meloidogyne incognita*	styrene	Luo et al. [126]
*B. subtilis* Bs-1	*Meloidogyne incognita*	CO_2_ acetic acid 2-heptanone pyrazine, 2,5-dimethyl- dimethyl disulfide	Cao et al. [125]
*Bacillu*s sp. GBSC56	*Meloidogyne incognita*	dimethyl disulfide methyl isovalerate 2-undecanone	Ayaz et al. [131]
*B. cereus* Bc-cm103	*Meloidogyne incognita*	dimethyl disulfide S-methyl ester butanethioic acid	Yin et al. [130]
*B. licheniformis* JF-22	*Meloidogyne incognita*	acetoin 2,3-butanediol hexamethyl cyclotrisiloxane	Du et al. [132]
*B. altitudinis* AMCC 1040	*Meloidogyne incognita*	2,3-butanedione acetic acid 2-isopropoxy ethylamine 2-methyl-butyric acid 3-methylbutyric acid octanoic acid	Ye et al. [133]
*Bacillus megaterium* YMF3.25	*Meloidogyne incognita*	benzeneacetaldehyde decanal dimethyl disulfide 2-nonanone 2-undecanone	Huang et al. [129]
*Bacillus aryabhattai* MCCC 1K02966	*Meloidogyne incognita*	dimethyl disulfide methyl thioacetate	Chen et al. [134]

## 5. Future Outlook on *Bacillus*-Based VOCs Research and Application

As presented in the previous sections, recent research on *Bacillus*-based VOCs has provided useful results in terms of (a) screening of *Bacillus* strains producing VOCs exhibiting antibacterial, antifungal, and nematicidal activity; (b) identification of the VOCs present in the volatile mixtures emitted by *Bacillus* spp.; (c) targeting the specific VOCs responsible for the antibacterial, antifungal, and nematicidal effects, as well as evaluation of the bacteriostatic/bactericidal and fungistatic/fungicidal concentrations of the specific VOCs; (d) *in vitro*, *in vivo*, and field testing of the specific VOCs/VOCs mixtures on the suppression of bacterial, fungal, and nematode plant pathogens. While the first studies in this field focused on identifying *Bacillus* strains producing VOCs with possible biocontrol applications, the research was later directed towards the precise identification of the produced VOCs and promotion of the ones responsible for biocontrol activity. Recent and currently ongoing studies are mostly focused on understanding the underlying mechanisms of antibacterial, antifungal, and nematicidal activity, both at the levels of cell structure and metabolism. However, a more profound understanding of the aforementioned mechanisms is required to maximize the potential of *Bacillus*-based VOCs biocontrol applications, leaving a significant space for further research in this area. Furthermore, *in vivo* testing procedures have been mostly based on model plants so far, requiring the widening of the host palette to increase the applicability and test the VOCs’ biocontrol potential in realistic application conditions, with more studies needed to be performed in greenhouses and in the field, followed by an investigation of the VOCs’ possible negative effects to the environment, animal- and human health. One of the possible research directions is also related to the biotechnological production of VOCs, directly affecting the mode of their biocontrol applications. Specific remarks related to all of the aforementioned aspects are given in the following subsections.

### 5.1. The Necessity to Investigate the Effects of Microbial Communities on Bacillus-VOCs Synthesis and Vice-Versa

Considering the previously mentioned fact that VOCs serve as signaling molecules for intra- and interspecies interactions as well as communication mediators across the kingdoms, it is necessary to better understand the exact mechanisms of microbial VOCs-mediated communication, considering the complexity of microbial communities in different ecological niches. So far, it has been revealed that VOCs help microorganisms make distinctions between neighboring microorganisms (friend, foe, or prey) and adjust their behavior and performance (persist, invade, escape, or defend) accordingly [11,135]. Furthermore, it is necessary to understand the mechanisms underlying volatile emission and perception, since it has been very challenging to detect the signal senders, receivers, and putative eavesdroppers [10]. Biodiversity is a key driver of several ecosystem functions [136], hence the interactions among the ecosystem members affect the production and function of VOCs. Diverse examples of shifts in VOC production by bacteria in the presence of other organisms involved in different communication pathways could be found so far, although this field is still under-investigated. An increase or decrease of the produced VOCs’ diversity and amount could be observed depending on the ecosystem community structure and the types of interactions, ranging from beneficial to antagonistic, resulting in the triggering/silencing of the VOCs’ production (mostly mediated by quorum sensing). Beneficial interactions result in additive/complementary/facilitative effects on VOC production and could arise from the induction of certain VOC production in the presence of specific strains/their metabolites, possibly acting as molecular inducers or precursors for VOC synthesis. On the other hand, antagonistic interactions are usually related to direct antagonism as well as the necessity to provide a competitive advantage in terms of growth space and nutrients in a limited ecological niche [7]. The aforementioned interactions in complex microbial communities could result in the production of novel VOCs that were not detected in the respective monocultures, and consequently, in a higher diversity of VOCs with distinct biological functions observed separately and together. One of the reasons for that could be the horizontal acquisition of the genes for the synthesis of volatile secondary metabolites by bacteria [3], which should be further investigated. Hence, there is a necessity to increase the diversity of the investigated microbial communities and shift from pure monocultures to more complex systems that include (micro)organisms representative of certain applications of the investigated VOCs. Although the soil communities are the most complex in terms of diversity and the number of present species, it is also important to direct investigations to the plant phyllosphere as well as to endophytic microbial interactions resulting in VOC production.

### 5.2. The Necessity to Better Understand the VOCs’ Mechanisms of Action against Broader Spectrum of Plant Pathogens and Hosts

Although a lot of literature data could be found regarding VOCs’ effect in terms of antifungal activity, their effects against other types of plant pathogens are still not sufficiently investigated. Furthermore, the majority of the studies deal only with the confirmation of antifungal/antibacterial/nematicidal activity of the mixture of VOCs produced by the certain *Bacillus* strain, while a minority of the studies involve a precise investigation of the activity of separate VOCs from the emitted mixture. There could be observed significant differences in terms of biocontrol efficacy between VOC blends (with specific concentrations and ratios or naturally produced) and single components, both in terms of general efficacy as well as in terms of the range of pathogenic targets. Currently, <10% of mVOCs have been assigned a function [13]. Taking into account overlaps in the biological roles of many VOCs as well as the raising evidence on different antimicrobial activities of chiral VOCs/enantiomers, there is a necessity for complete chemical characterization and molecular targets’ profiling of the VOCs in future investigations [10]. Furthermore, limited knowledge about the perception of microbial volatiles by other (micro)organisms is currently available. Therefore, further research should focus on mechanisms of VOC cell entry and cell targets, and here microbial mutants could offer a useful toolbox to detect genes responsible for the perception of VOCs, as well as their cellular/molecular targets [11]. In general, a greater understanding of VOCs’ mechanisms of action across a broader range of pathogenic microorganisms is required, besides the necessity to include a wider range of plant hosts going behind the model plant species, considering that most of the current research refers to *Arabidopsis thaliana* and *Nicotiana benthamiana* [13].

### 5.3. Research Directions Related to VOCs Production by Bacillus spp.

VOCs’ potential application is directly related to the selected VOC source—pure compounds, *Bacillus*-based captured VOCs, or the use of viable cells for on-site VOC production and application. Some of the VOCs produced by *Bacillus* strains could be chemically synthesized and used as pure compounds in different modes of application. On the other hand, VOCs produced by *Bacillus* spp. could be captured in a closed system and used for plant pathogen suppression at the same or a different location. Moreover, simultaneous production and application of *Bacillus*-based VOCs could be achieved, either by direct inoculation of biocontrol strains to plants or the surrounding soil or by maintaining cultures producing VOCs in plants’ close vicinity, which would be the suitable mode of application in closed and controlled systems, such as greenhouses. Independently of the application mode, it is necessary to consider the main bioprocess variables affecting VOC production by *Bacillus* strains. On the upstream side of the bioprocess, the main bioprocess development step is the selection of suitable nutrients to be included in the solid or liquid cultivation medium, depending on the metabolic pathways required for the production of targeted VOCs and the substrates required in these metabolic processes, as well as optimization of the medium pH value as one of the most important physiology-affecting parameters. The next step is to optimize nutrients’ ratios and concentrations to maximize target VOC production, as well as to decide between the utilization of solid or liquid medium, which significantly affects other bioprocess variables, such as mass and oxygen transfer. This leads us to the cultivation step itself, where it is necessary to determine the production system design, significantly depending on the previous selection between solid and liquid cultivation medium, and to optimize the cultivation parameters (inoculum preparation and inoculation strategy, medium volume, T, mixing and aeration rate, duration) to minimize resource utilization and achieve cost-effectiveness in terms of energy consumption. Furthermore, downstream and analytical methods require constant advancements to collect and detect the produced volatiles correctly, especially in cases where the collected volatiles are further used as biocontrol agents. Most of the currently available studies deal with VOCs produced by a single strain cultivated on artificial nutrient-rich media [137], not taking into account the real conditions where the VOCs will be applied, both in terms of the previously mentioned problems related to the diversity of natural microbial communities and environmental conditions. Hence, future research should focus on the imitation of environmental conditions at the VOC application site in terms of nutrient supply, physico-chemical properties of the soil, and climate factors shaping the growth and development of the bacteria but also their volatile emission [3]. The factors that should be taken into account include soil aeration and limited oxygen availability in the rhizosphere, together with regional, seasonal, or climate dependencies and nutritional profiling in terms of the dependency of microbial growth and metabolism on root exudates. On the other hand, poor soil nutrient status is a raising trend in different geographic regions across the globe. Simulation of VOCs application environmental conditions should be implemented into the general bioprocessing strategy, starting from the cultivation medium whose composition strongly affects VOCs profile and amount and choice between solid and liquid media, mostly affecting oxygen availability [138]. Furthermore, different cultivation conditions (T, pH, mixing and aeration rates, duration) should be investigated to predict the VOC emission in a real-world application environment as well as to maximize their efficiency in terms of optimal diversity and concentration. Another important aspect for future investigations includes the monitoring and analysis of volatile secondary metabolites related to specific stages of bacterial growth and development, especially in terms of multicellular behaviors of *Bacillus* spp., including motility of freely available cells, sporulation, and biofilm formation [3].

### 5.4. VOCs Application-Related Remarks

Recent research of *Bacillus*-based VOCs has revealed varying differences in their effects from the lab to the field, mostly due to significant differences between in vitro production and testing conditions and real application conditions, including abiotic soil conditions (temperature, pH, moisture content, and soil texture), as well as the availability and quality of organic energy sources [137]. It points out the requirement for more field studies in terms of VOCs efficacy, which are currently scarce. Considering that the exact VOC concentration in the complex volatile mixture emitted by *Bacillus* spp. is usually not determined, it is very important to incorporate a wider concentration range in the testing protocols as many VOCs reveal dose-dependent biocontrol effects. The perception of a microbial VOC by other (micro)organisms could be related to its ratio/concentration in the complex volatile mixture, besides its presence. Therefore, this complex background should be incorporated as much as possible in the experimental setups designed to assess the biological activity of VOCs, facilitating the transition from laboratory research to real-life application conditions [10]. The incorporation of sensors for continuous monitoring of VOC concentration and big data computing analytics represent promising auxiliary tools for sustainable agricultural practice [2]. *Bacillus*-based VOCs could be applied, e.g., as repellents or biopesticides against plant diseases caused by microbial pathogens, soil fumigants, and fumigants in the management of post-harvest diseases. Their mode of use depends a lot on how far these VOCs travel before they cause a biological response in other organisms [11]. It opens a chapter of possibilities for VOCs delivery, including injection, dripping, drench versus spray application, companion cropping systems, etc. [13]. Utilization of different carriers for immobilization of *Bacillus*-producing VOCs, which could be supported by, e.g., 3D bioprinting, is worthy of investigation in future studies. One of the possible investigation routes includes the utilization of organic soil amendments that promote the production of VOCs during decomposition, where the necessity of the presence of VOC precursors in organic amendments should be further examined [137].

### 5.5. Possible Risks of Bacillus-Based VOCs Application in Biocontrol of Plant Diseases and Pathogens

Due to their biological origin, *Bacillus*- and other microbially-derived VOCs are usually perceived as safe for the environment, plants, animals, and humans due to their lower (eco)toxicity and biodegradability. Some studies have observed the phytotoxic effects of higher VOC concentrations at different plant growth stages. Hence, there is a necessity to optimize VOC dosage, delivery, and time of treatment. Furthermore, when applying a mixture of VOCs emitted by a certain *Bacillus* strain present directly on the plant or in its close vicinity, it is necessary to investigate the strain’s overall capacity for VOC production depending on the growth conditions, i.e., to predict the strain’s volatile profile expected under certain application conditions to prevent the emergence of (phyto)toxic volatiles. Moreover, it is necessary to predict the strain’s volatile profile independently of the growth conditions by knowing and understanding the strain’s genetic basis for VOC production, where whole genome sequencing technology could be useful together with databases of genomic data on *Bacillus* strains capable of VOC production to provide the basis for the selection of safe strains in terms of possible VOC release risks. Due to high evaporation rates and a lack of stability, VOC application may require high initial concentrations, raising the risk of dose-dependent toxicity against plants and other non-target organisms [137]. Dimethyl disulfide, produced by many *Bacillus* strains, is commercialized as one of the most potent microbial VOCs, exhibiting antifungal and nematicidal action [139,140]. However, recent studies have reported possible toxicity-related issues, including potential eye- and skin-irritable properties during acute exposure, while exposure to large concentrations may cause nausea, headache, dizziness, and irritation of the upper respiratory tract [141]. Therefore, besides the utilization of VOCs as pure compounds, which have been previously proven to be safe for humans and animals, the toxicity and non-target effects of the VOCs need to be documented before any field application, including effects on mycorrhizal development, non-target beneficial soil organisms, and a possible increase in other harmful soil organisms, to overcome the translational gap related to *Bacillus*-VOCs unexpected effects [10,11,137].
